# Temporal and spatial changes in ion homeostasis, antioxidant defense and accumulation of flavonoids and glycolipid in a halophyte *Sesuvium portulacastrum* (L.) L.

**DOI:** 10.1371/journal.pone.0193394

**Published:** 2018-04-11

**Authors:** Ganesh C. Nikalje, P. S. Variyar, M. V. Joshi, T. D. Nikam, P. Suprasanna

**Affiliations:** 1 Department of Botany, Savitribai Phule Pune University, Pune, India; 2 Nuclear Agriculture and Biotechnology Division, Bhabha Atomic Research Centre, Trombay, Mumbai, India; 3 Department of Botany, R.K. Talreja College of Arts, Science and Commerce, Ulhasnagar, Thane, India; 4 Food Technology Division, Bhabha Atomic Research Centre, Trombay, Mumbai, India; 5 National Facility for High-field NMR, Tata Institute of Fundamental Research, Mumbai, India; Huazhong Agriculture University, CHINA

## Abstract

Salinity is an important environmental constraint limiting plant productivity. Understanding adaptive responses of halophytes to high saline environments may offer clues to manage and improve salt stress in crop plants. We have studied physiological, biochemical and metabolic changes in a perennial, fast growing halophyte, *Sesuvium portulacastrum* under 0 mM (control), 150 mM (low salt, LS) and 500 mM (high salt, HS) NaCl treatments. The changes in growth, relative water content, cation, osmolyte accumulation, H_2_O_2_ and antioxidant enzyme activity (SOD, CAT and APX) were observed under different treatment conditions. A positive correlation was revealed for sodium ion accumulation with malondialdehyde (r^2^ = 0.77), proline (r^2^ = 0.88) and chlorophyll content (r^2^ = 0.82) under salt treatment while a negative correlation was observed with relative tissue water content (r^2^ = -0.73). The roots and leaves showed contrasting accumulation of potassium and sodium ions under LS treatment. Temporal and spatial study of sodium and potassium ion content indicated differential accumulation pattern in roots and leaves, and, high potassium levels in root. Higher H_2_O_2_ content was recorded in roots than leaves and the antioxidant enzyme activities also showed significant induction under salt treatment conditions. Gene expression profiling of sodium transporters, *Sodium proton exchanger* (*NHX3*), *Vacuolar ATPase* (*vATPase*) and *Salt overly sensitive1* (*SOS1*) showed up regulation under salt stress after 6–24 hr of NaCl treatment. Metabolite changes in the salt stressed leaves showed increased accumulation of flavonoids (3,5-dihydroxy-6,4’-dimethoxy-flavone-7-O-[α-L-rhamnopyranosyl-(1→6)-β-D-glucopyranoside], and3,5-dihydroxy-6,3’,4’-trimethoxy-flavone-7-O-[α-L-rhamnopyranosyl-(1→6)-β-D-glucopyranoside] in both LS and HS treatments, while a glycolipid, 1-O-linolenyl-2-O-(palmitoyl)-3-O-galactopyranosyl glycerol, accumulated more in LS over HS treatments and control. The results suggest that differential spatial and temporal cation levels in roots and leaves, and accumulation of flavanoid and glycolipid could be responsible for salt adaptation of *S*. *portulacastrum*.

## 1. Introduction

Soil salinity is one of the most serious causes of reduced crop productivity [1]. Salinity causes physiological, biochemical, and morphological alterations through osmotic and ionic imbalance in plants [2, 3]. Being sessile, plants have developed strategies to withstand against salinity stress which include compartmentalization of salt ions, synthesis of compatible solutes, antioxidant enzymes, osmolytes, hormone modulation and secondary metabolites [4, 5, 3]. Salinity induced reactive oxygen species (ROS) such as hydrogen peroxide (H_2_O_2_) and superoxide anion are toxic and hence, antioxidant machinery is activated in plants mitigates ROS induced cellular damage [6, 7, 8]. ROS has been shown to impact ion fluxes in Arabidopsis roots under salt stress via the activation of outward rectifying K^+^ channels and K^+^ loss [9]. Several reports have indicated that salt tolerance responses constitute efficient osmotic fine-tuning in terms of reduced sodium uptake and favored potassium retention, tissue-specific sodium sequestration, and minimal oxidative damage [10, 11]

On the basis of salt tolerance ability, plants are categorized as halophytes and glycophytes. Glycophytes are salt sensitive and hence, high sodium content in soil affects every aspect of plants growth, development and metabolism. Halophytes are the native flora of saline soil with exceptional ability to tolerate high salinity. They grow and complete their life cycle on a salt rich substratum [12]. These plants are either salt excluders that simply do not allow toxic salt ions to enter in to xylem stream or they take up salt ions and extrude them from leaf with help of specialized structures such as salt glands or salt hairs. On the other hand, salt accumulators absorb salt ions through their roots and sequester them in to leaf vacuole. In order to survive under high saline environment, halophytes have to efficiently manage ion homeostasis through transporters [13] and sodium sequestration in vacuole [5]. Under saline conditions, halophytes use Na^+^, Cl^-^, and K^+^ for maintenance of shoot osmotic pressure which in case of glycophytes is managed through synthesis of compatible solutes [10, 14]. It has also been shown that these inorganic ions constitute 80–95% of the osmotic pressure of the cell sap in halophytes, while in non-halophytes it ranges between 50 to 70% [15]. Other compatible solutes like proline, glycine betaine, sugars, phenolics and, antioxidant system comprising of antioxidant enzymes (superoxide dismutase, catalase, guiacol peroxidase, ascorbate peroxidase, glutathione reductase) and non-enzymatic antioxidants (like ascorbate, glutathione) also play an important role in the adaptation mechanism of halophytes [5].

Activation of ion transporters such as *SOS1* and *NHX1* has been suggested to passively regulate Na^+^ flux [16]. Some halophytes rapidly absorb Na^+^ ions from the root zone and transport them to aerial parts where it is sequestered in to vacuoles. During this process, ion transporters like sodium proton exchanger (*NHX)*, salt overly sensitive (*SOS*), tonoplast localized ATPases (*v-type ATPase*) play a vital role [15, 17]. The *NHX* family genes mediate coupled exchange of Na^+^ and H^+^. The vacuolar *NHXs* play an important role in compartmentalizing Na^+^ from cytosol to vacuole by exchanging cytosolic Na^+^ with vacuolar H^+^. The Salt Overly Sensitive 3 (*SOS3*) gene encodes myristoylated calcium binding protein, which acts as calcium sensor under salt stress and binds with *SOS2*, which is serine/threonine protein kinase to form a complex. This complex then activates *SOS1*, which acts as a Na^+^/H^+^ antiporter and excludes Na^+^ out of cytoplasm [8, 18]. High level of *SOS1* expression has been observed in salt accumulating halophytes (*Salicornia dolichostachya* and *Thellungiella halophila*), [19, 20]. The *v-type ATPase* generates proton motive force for the compartmentalization of sodium ions into vacuole [21]. Under salt stress, the increased activity of vacuolar H^+^ pumps was shown to coordinate with vacuolar Na^+^/H^+^ antiporter for cation exchange [21]. In *Suaeda salsa*, higher induction of V-ATPase and Na^+^/H^+^ antiporter activities was observed in tonoplast vesicles under salinity [22]. Such a salt-dependent increased activity upon NaCl addition has also been observed in *Mesembryanthemum crystallinum*[23].*Salicornia bigelovii*[24] and *Lobularia maritime*[25]. Induction of both *SOS1* and *NHX1* in leaf and root tissues of a perennial halophyte, *Aeluropus littoralis* was suggested to be effective to control Na^+^ translocation and accumulation in leaf tissues [26].

Plants synthesize a number of specific metabolites to adapt to abiotic stresses [27]. Analysis of biochemical composition and metabolites under stress conditions can throw light on the synthesis of new bioactive compounds of relevance to stress physiology of the plant [28]. Most of the metabolic profiling protocols utilized methanol for the identification of differentially accumulated metabolites under specific environmental conditions [29]. The accumulated metabolites are different in terms of polarity, chemical behaviour, stability and concentration [29]. Therefore, a single solvent or extraction protocol may not give comprehensive coverage of total metabolites. In response to abiotic stresses, plants respond by modulating their metabolic activities [30]. Specific response to salt stress adaptation and tolerance in some halophytes has been related to accumulation of flavonoids, sulpholipids, polyphenols [31, 32]. These preliminary metabolomic studies have been useful to draw basic map of chemical signatures or metabolites associated with salt adaptation.

S*esuvium portulacastrum*, a member of Aizoaceae family, is a perennial halophyte with medicinal properties, aesthetic value and it produces industrially important ecdysones besides having significant tolerance to heavy metals [33]. This plant has good potential to be used for desalination of salt-affected soils or reclamation of saline soil [34, 35]. In our earlier studies, we have shown that *Sesuvium* exhibits better photosynthetic activity, succulence, biomass and antioxidant defense mechanisms at cellular, organ and plant level [3, 33]. Stress induced lipid peroxidation and relative electrolyte leakage, which are considered as stress biomarkers have also been shown to designate salt-induced damage at higher NaCl concentrations [36]. Higher levels of proline and glycine betaine, total soluble sugars were reported as a mark of osmotic adjustment in *Sesuvium* grown at 200 and 500 mM NaCl [36, 37]. These studies have mostly demonstrated salt responsive physiological effects, however information is limited on salinity induced ionic perturbations, expression profiling of transporter genes and metabolic alterations. With this intent, we have studied low and high salt stress induced changes at growth, biochemical and metabolic levels in *Sesuvium portulacastrum*, and presented results on the tissue specific accumulation of sodium and potassium ions, osmolytes and antioxidant enzymes in root and leaf tissues, increased expression of sodium transporters[*Sodium proton exchanger* (*NHX3*), *Vacuolar ATPase* (*vATPase*)and *Salt overly sensitive1* (*SOS1*)], correlation of sodium ions with stress indicators and accumulation of a flavonoid and glycolipid under varying salt regimes which may play a crucial role in salt adaptation mechanism of *Sesuvium portulacastrum*.

## 2. Material and methods

### 2.1 Plant material and salt treatments

The axillary shoots of *Sesuvium portulacastrum* L. were collected from the coastal plains of Vashi, Navi Mumbai, India. The shoots were surface sterilized with 1% Bavistin containing few drops of Tween-20 for five minutes and subsequently washed with distilled water to remove chemical traces. This was followed by 0.1% mercuric chloride treatment for 1 minute and rinsing for five times with sterile distilled water. The surface sterilized, 5–6 cm long shoots with two nodes and two opposite leaves were grown in half strength Hoagland’s medium under hydroponic conditions. The well-rooted shoots with 4–6 fully expanded leaves (hereafter referred to as plants) were grown at 25 ± 2°C under 16 hr photoperiod (30 µmol.m^-1^.s^-1^ PFD) using cool white fluorescent tube lights (Philips Ltd, Mumbai, India) and humidity maintained at >60%. After four weeks, well rooted plants were subjected to 0 (control, No salt), low salt (150 mM, LS) and high salt (500 mM, HS) treatments in half strength Hoagland’s medium and losses due to evaporation were replenished every alternate day with the respective strength of salt solutions. The plants were harvested after 28 days of salt treatment for all the studies including physiological, biochemical and ion accumulation. For temporal and spatial expression analysis study, plants were harvested after 2hr, 6hr, 24hr, 3d, 5d and 7d and, for expression analysis of transporter genes, plants were harvested after 2hr, 6hr, and 24hr post NaCl treatment.

### 2.2 Growth and chlorophyll measurements

Plant growth was assessed based on the changes in fresh weight, and root and shoot length. After harvesting, roots were washed five times with chilled distilled water, so as to remove the traces of excess salt which adhered on the root surface. The plants were then blot dried between two layers of tissue paper and the shoot and root tissue were separated followed by oven drying at 60°C for 48h. The chlorophyll content (chlorophyll a, chlorophyll b and total chlorophyll) was estimated by the method of Arnon [38] and Al Hassan et al. [39]. The chlorophyll pigments were extracted in 80% chilled acetone in dark and centrifuged at 12,000 rpm for 10 min at 4°C. The absorbance was taken at 663 and 645 nm.

### 2.3 Relative water content (RWC)

The relative water content was estimated as per Diaz-Perez et al. [40]. The leaves from the treated and untreated plants were harvested to record fresh weight (FW), and were then immediately placed in screw cap tubes containing 10 ml of distilled water and incubated on shaking incubator (180 rpm) at 25ºC for 24 h. Turgor weight (TW) was recorded after blotting the tissue on paper towel and the tissue was oven dried at 80ºC for 48 hr to measure dry weight (DW). RWC was calculated by using the following formula:

RWC (%) = [(FW-DW) / (TW-DW)] X 100

### 2.4 MDA content

Lipid peroxidation was determined by the estimation of malondialdehyde (MDA) content following Hodges et al. [41] and [42]. Plant material (500 mg) was homogenized with 3 ml of 0.5% thiobarbituric acid in 20% trichloroacetic acid (w/v). The homogenate was incubated at 95°C for 30 min and reaction was stopped on ice. The samples were centrifuged at 10,000×g for 10 min and absorbance was recorded at 532 and 600 nm (UV–1700 PharmaSpec, UV-visible spectrophotometer, Shimadzu, Japan). The amount of MDA (extinction coefficient of 155mM^−1^ cm^−1^) was calculated by subtracting non-specific absorbance at 600 nm from absorbance at 532 nm.

### 2. 5 Osmolytes accumulation

#### 2.5.1 Proline

Proline content was determined according to the method of Bates et al. [43] and Al Hassan et al. [39]. The 100 mg fresh tissue was ground in liquid nitrogen and homogenized in 3% sulfosalicylic acid. The homogenate was centrifuged at 10,000 rpm for 10 min at 4°C. The supernatant (0.5 ml) was then treated with 0.5 ml each with acid ninhydrin reagent and glacial acetic acid. The reaction mixture was incubated at 100°C for 1 hr in water bath. The reaction was terminated by keeping the tubes on ice bath and allowed to cool at room temperature. Toluene (2 ml) was added to reaction mixture and mixed vigorously for 15 seconds. The chromophore group containing proline was aspirated and optical density was taken at 520 nm (UV–1700 PharmaSpec, UV-visible spectrophotometer, Shimadzu, Japan). The amount of proline was determined from a standard curve using L-proline and was expressed as µg of proline g^-1^ FW.

#### 2.5.2 Total soluble sugar

TSS was estimated as per the anthrone method [44] with some modifications. About 200 mg of fresh tissue was homogenized in ice-cold 80% ethanol using mortar and pestle. The extract centrifuged at 5,000 rpm for 10 min at 4°C and final volume was adjusted to 10 ml with 80% ethanol. Then 1.0 ml of supernatant was reacted with 3.0 ml of freshly prepared anthrone reagent by incubating the reaction mixture for 10 min at 100°C in hot water bath. The reaction was terminated by quick cooling in an ice bath and allowed to cool at room temperature. The optical density was measured spectrophotometrically at 620 nm. A standard curve was prepared using D-glucose; the TSS was calculated and expressed as mg g^-1^ FW.

### 2.6 Determination of Na^+^, K^+^ and Ca^2+^

For temporal and spatial study of ion accumulation, plants were harvested after 2hr, 6hr, 24hr, 3d, 5d and 7d of salt treatment and for rest of the experiments, plants were harvested after 28 days of salt treatment. For cation analysis, dried root and leaf samples (50 mg DW) were incubated overnight in concentrated nitric acid (HNO_3_) and digested at 150°C for 30 min. The digested samples were then dissolved in 10 ml deionized distilled water. Ion content was analyzed by microcontroller flame photometer (Labtronics, model no-LT-671, India) as per manufacturer’s instructions.

### 2.7 Hydrogen peroxide (H_2_O_2_)

The H_2_O_2_ content was determined as earlier described by Alexieva et al. [45]. About 100 mg tissue was homogenized in 0.5% (w/v) trichloroacetic acid and centrifuged at 14,000x g at 4°C for 15 min. The 0.5 ml supernatant was mixed with 0.5 ml 100 mM potassium phosphate buffer (pH 7.0) and 1 ml freshly prepared 1M potassium iodide. The reaction was incubated in dark for 1 hr and absorbance was measured spectrophotometrically at 390 nm. A Standard curve was prepared using known concentration of H_2_O_2_.

### 2.8 Antioxidant enzymes assay

Plant material (500 mg) was homogenized in 100 mM pre-chilled potassium phosphate buffer (pH 7.0) containing 0.1mM EDTA and 1% (w/v) polyvinyl pyrolidone. The homogenate was squeezed through four layers of cheese cloth and extract thus obtained was centrifuged at 15,000×*g* for 15 min at 4°C. The resulting supernatant was used to measure the activities of SOD and APX. The protein content in the supernatant was measured according to Lowry et al. [46].

The activity of superoxide diamutase (SOD) was assayed as per the method described by Beauchamp and Fridovich [47] by measuring enzyme ability to inhibit the photochemical reduction of nitroblue tetrazolium (NBT). The 3ml reaction mixture contained 40mM phosphate buffer (pH 7.8), 13mM methionine, 75 µM NBT, 2µM riboflavin, 0.1mM EDTA and a suitable aliquot of enzyme extract. The test tubes were shaken and placed 30 cm below light source consisting of 15W fluorescent lamp. The absorbance was taken at 560 nm. The activity of SOD was expressed as units.mg^−1^ protein. One unit of activity is the amount of protein required to inhibit 50% initial reduction of NBT under light.

Ascorbate peroxidase (APX) activity was measured by estimating the rate of ascorbate oxidation (extinction coefficient 2.8mM^−1^cm^−1^). The 3ml reaction mixture contained 50mM phosphate buffer (pH 7.0), 0.1mM H_2_O_2_, 0.5mM sodium ascorbate, 0.1mM EDTA and a suitable aliquot of enzyme extract. The change in absorbance was monitored at 290 nm [48] and enzyme activity was expressed as units mg^−1^ protein.

For the measurement of catalase (CAT) activity, extraction was done in the buffer containing 50mM Tris–HCl (pH 7.0), 0.1mM EDTA, 1mM PMSF and 0.3 g g^−1^ fw PVP. Activity was measured by the method of Aebi [49]. The 3ml reaction mixture comprised of 50mM sodium phosphate buffer (pH 7.0), 20mM H_2_O_2_ and a suitable aliquot of enzyme. Decrease in the absorbance was taken at 240 nm (molar extinction coefficient of H_2_O_2_ was 0.04 cm^2^ mol^−1^). Enzyme activity was expressed as units mg^−1^ protein.

### 2.9 Gene expression profiling of Na^+^ related transporters

Three sodium transporters *Sodium proton exchanger* (*NHX3*), *Vacuolar ATPases* (*vATPase*) and *Salt overly sensitive1* (*SOS1*) were selected for expression profiling study by using real time (qRT) PCR. Both root and leaf tissues were harvested at 2hr, 6hr and 24hr post NaCl treatments and RNA was isolated by using miRVANA plant RNA isolation kit (AM1560) as per the manufacturer’s instructions. The integrity and quality of RNA was checked by electrophoresis of total RNA on 1.2% agarose gel and measuring the ratio of absorbance at 260 and 280 (A260/280).

#### 2.9.1 Primer designing

Primers were designed from the conserved regions of *Mesembryanthemum crystallinum* and *Arabidopsis thaliana* by using Primer 3 software. Primers were synthesized from Metabion International (Germany; www.metabion.com/). Details of primers, primer sequences and amplicon length are given in S1 Table.

#### 2.9.2 Real-time PCR

The DNA-free intact RNA (3µg) was subjected to cDNA synthesis using a Stratagene high fidelity first-strand cDNA synthesis kit, as per the manufacturer’s instructions (www.stratagene.com/). The cDNAs were then stored at –20 ^o^C until used for real-time PCR. Real-time PCR was carried out using a Corbett RotorGene 6000 (Corbett Life Science, www.corbettlifescience.com/). The *S*. *portulacastrum Tubulin* gene was amplified in parallel with other target genes, for normalization of expression and quantification. *Tubulin* was verified to check for its level under given treatments before using it as a reference gene [50] The PCR efficiency of *Tubulin* and other genes was also checked which was approximately 1.96 to 1.99. SyBr green master mix kit (S 4320, Sigma) was used for qRT–PCR as per the manufacturer’s instructions. Approximately 2.5 µg cDNA was used as the template. The PCR cycle comprising of 95°C for 30 s, 55°C for 45 s, and 72°C for 30 s, PCR was run for 40 cycles with slight modification for some of the genes. For each sample, reactions were set up in triplicate to ensure reproducibility of the results.

### 2.10 Extraction, isolation and identification of metabolites

Leaf tissue from control, LS and HS treatments were harvested and lyophilized at -50°C for 24 hrs. About 100 g lyophilized leaf tissue from each treatment was extracted successively with hexane, chloroform and methanol using pressurized solvent extractor (Buchi). All the extracts were evaporated to dryness in a soxhlet extractor (at 30°C) and 10% solution of each extract was prepared in the respective extracting solvent. The extracts were subjected to analytical silica gel TLC using toluene: ethanol: formic acid (5:4:1) as the developing solvent. The separated spots were visualized by staining under iodine vapors. The differential spots from different solvent extracts were identified and taken up for further investigation.

The methanol extract was further subjected to silica gel column chromatography. The major compounds in the extract were isolated by successive elution with increasing proportion of methanol: water (100 ml each) (100: 0, 90:10, 80:20, 70:30, 60:40 and 50:50 v/v). The 10 ml of each elute collected were concentrated to 0.5 ml and then analyzed separately on TLC plate. The elutes with same R_f_ value were pooled together to obtain three major fractions containing the purified compounds. The purified compounds were identified by using NMR, UV-Vis spectrophotometer, FT-IR and ESI-MS. Details of the instrumentation used and operating conditions are given as supplementary information (S1 Info).

### 2.11 Statistical analysis

All the experiments were carried out in a randomized block design. Minimum three biological and two technical replicates were kept for each experiment and each replicate consisted of four 30 days old seedlings. One-way analysis of variance (ANOVA) was performed with all the data to confirm the variability of data and validity of results, and Duncan’s multiple range test (DMRT) was performed to determine the significant difference between treatments. Correlation analysis was performed for all the data for each parameter with respect to different salt treatment or between parameters (p < 0.05).

## 3. Results

### 3.1 Effect of NaCl on growth, RWC and chlorophyll pigments

**Fig 1:**
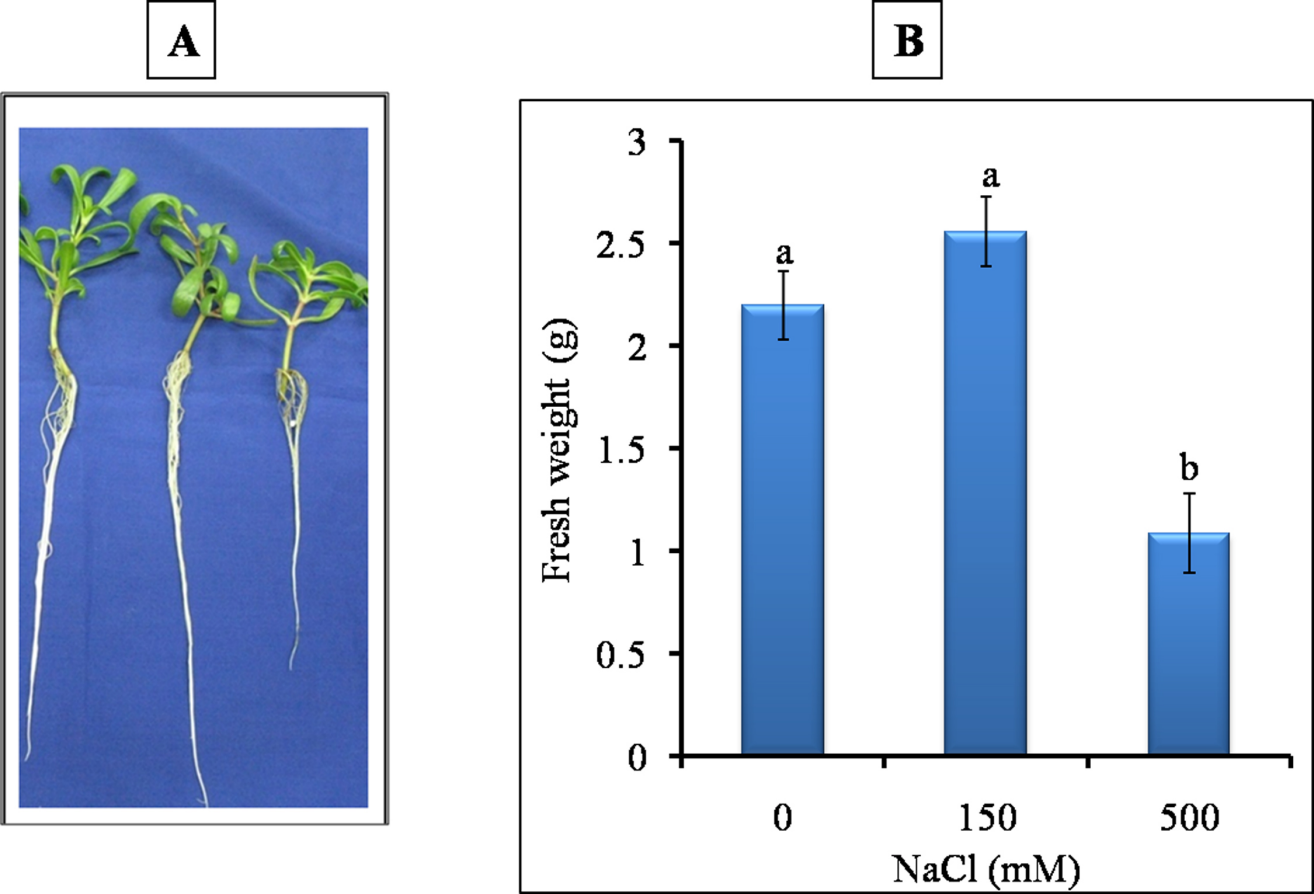
A) Effect of different NaCl treatments on growth of *S*. *portulacastrum*. C-control 0 mM NaCl), LS-Low salt 150 mM NaCl), HS-High salt 500 mM NaCl). B) Effect of salinity treatments on fresh weight of *S*. *portulacastrum* plants. Different letters over bar indicates significant difference between treatment according to Duncan’s multiple range test *p*≤ 0.05).

**Fig 2:**
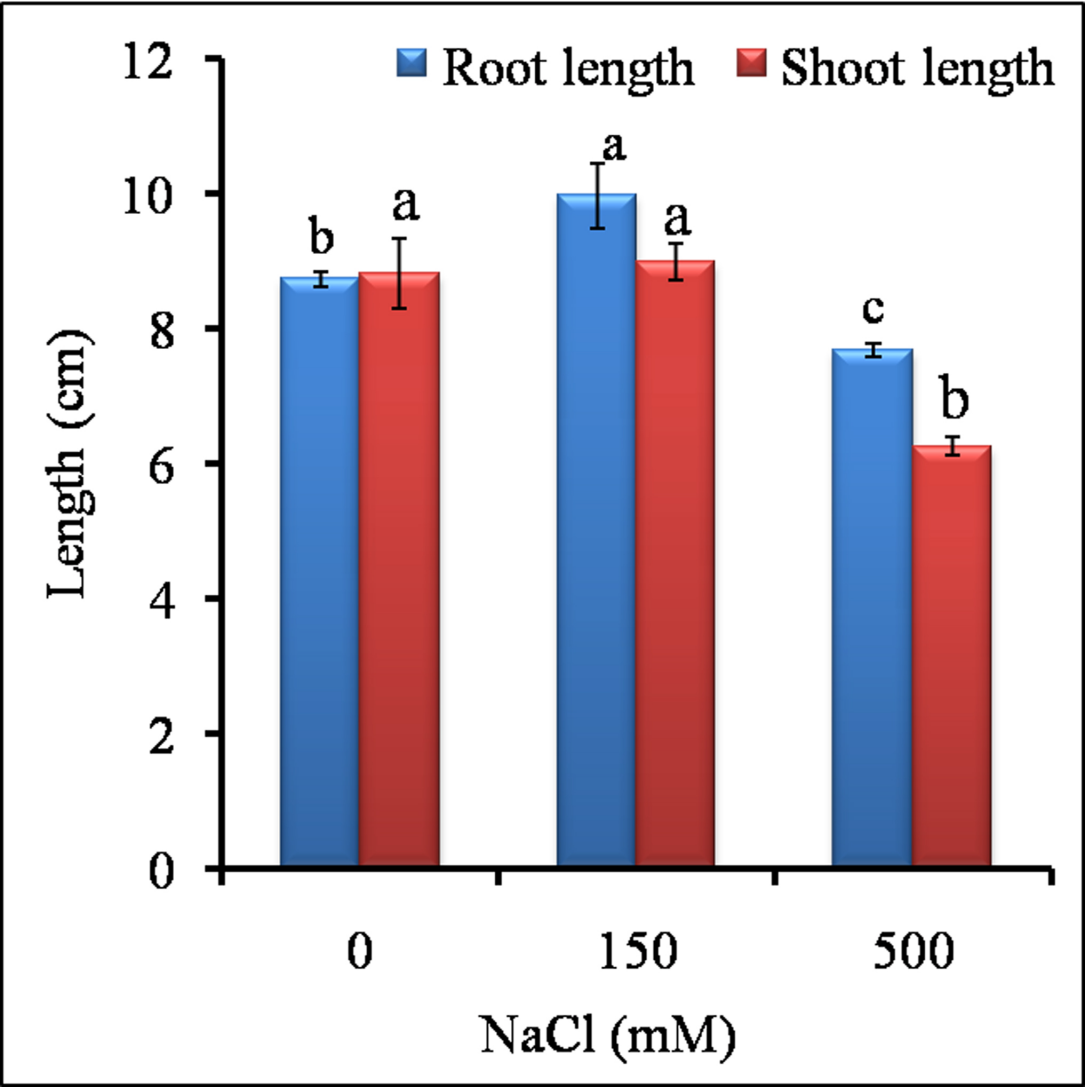
Effect of different NaCl treatments on root and shoot length of *S*. *portulacastrum* plants. Different letters over bar of same color indicates significant difference in treatment according to Duncan's multiple range test DMRT *p*≤ 0.05).

*Sesuvium* plants exposed to 0, 150 and 500 mM NaCl stress exhibited differential growth response (Fig 1). Salt stress with 150 mM NaCl (low salt treatment, LS) didnot show any negative effect on plant growth (Fig 1A and 1B) whereas growth of plants was significantly affected at 500 mM NaCl (high salt treatment, HS), (Fig 1A). The growth of HS treated plants in terms of fresh weight, root and shoot length was significantly lower than control and LS treated plants (Fig 1B). The LS treated plants showed significant increase in root length (by 1.1 fold) over control (Fig 2).

**Fig 3:**
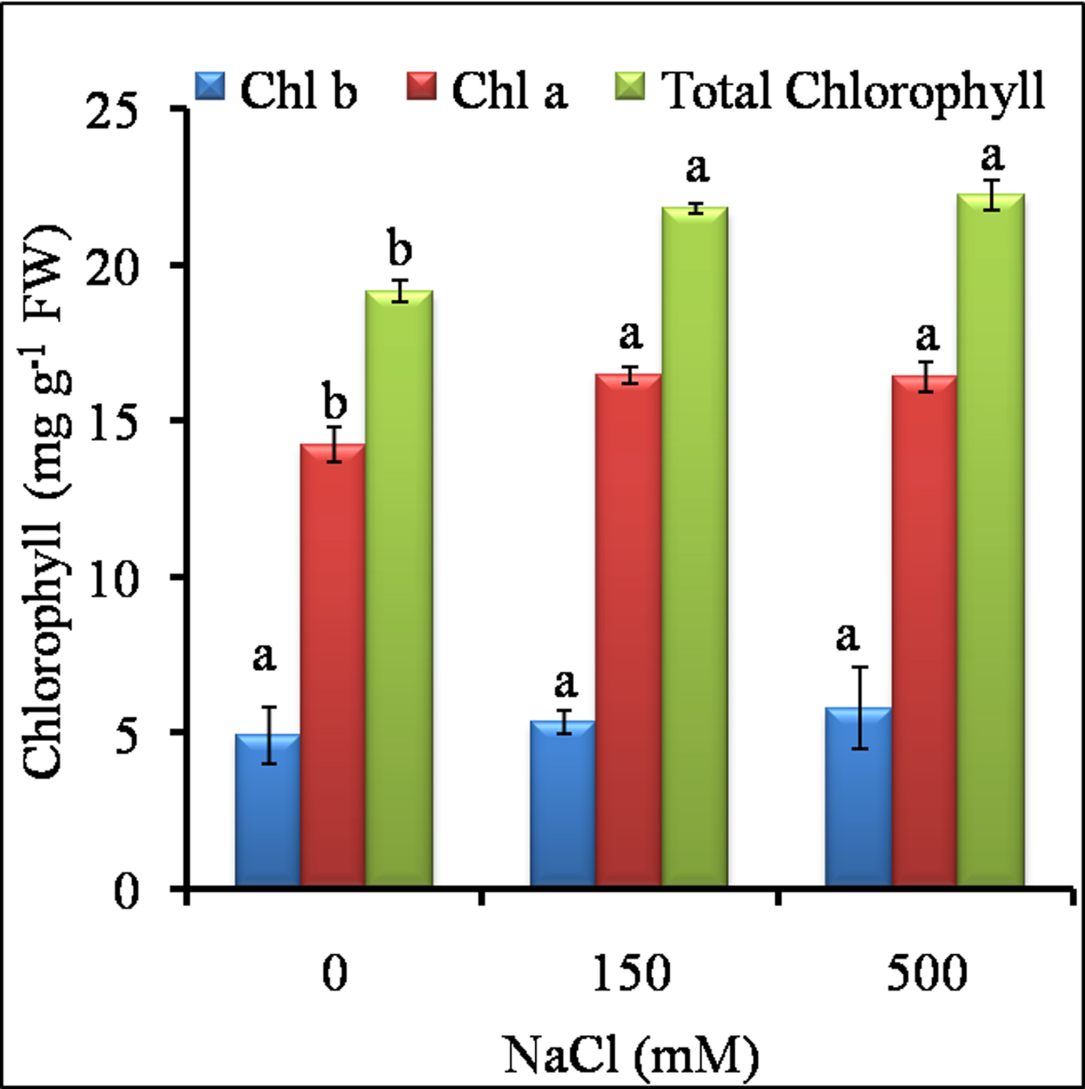
Estimation of chlorophyll pigments total chlorophyll, chlorophyll ‘A’ and chlorophyll ‘B’) under different NaCl treatments in *S*. *portulacastrum*. Different letters over bar with same color indicates significant difference in treatment according to Duncan's multiple range test DMRT *p*≤ 0.05).

**Fig 4:**
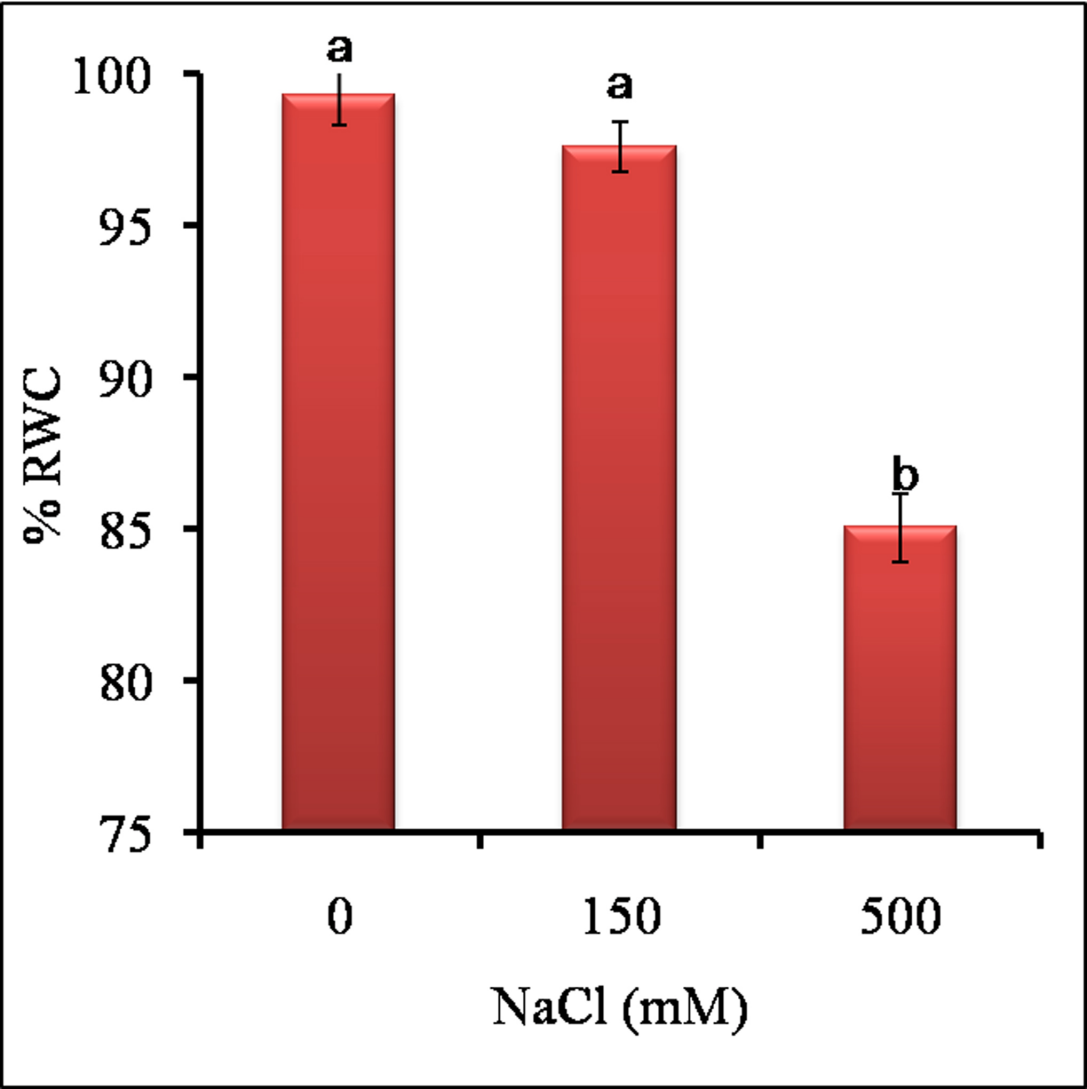
Effect of different NaCl treatments on % relative water content RWC) of leaves of *S*. *portulacastrum*. Different letters over bar indicates significant difference between treatments according to Duncan’s multiple range test *p*≤ 0.05).

In both the salt treatments, leaves remained green with significant increase in total chlorophyll and chlorophyll-content, whereas chlorophyll-b content was unaffected as compared to control (Fig 3). The % Relative water content (RWC) was almost stable at LS (150 mM) as the difference between control and LS was insignificant (Fig 4). At HS (500 mM), RWC decreased significantly but remained close to 85% as compared to control and LS. The results indicated that 150 mM was -favorable for growth of *Sesuvium* as there was stable RWC, no salt sensitivity and no growth reduction whereas, higher salinity (500 mM) affected growth with reduction in RWC.

### 3.2 MDA content

**Fig 5:**
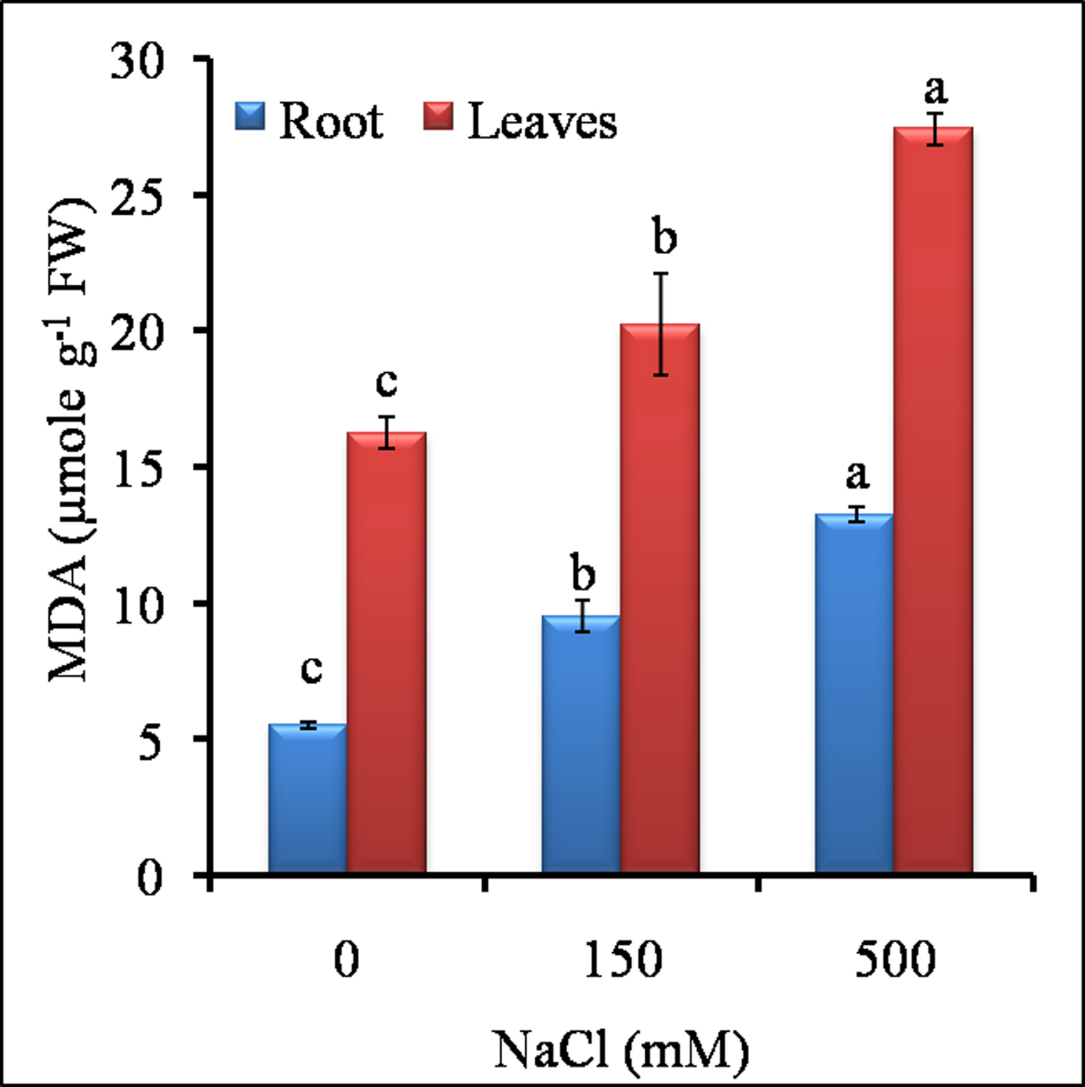
Effect of different NaCl treatments on MDA content of roots and leaves of *S*. *portulacastrum*. Different letters over bar with same color indicates significant difference in treatment according to Duncan's multiple range test DMRT *p*≤ 0.05).

MDA content was gradually increased with increment in salt concentration in both roots and leaves (Fig 5). In roots, LS and HS induced 1.6 and 2.4 fold higher MDA content respectively, than the control. In leaves, a similar trend observed with 2 fold higher MDA content in HS over LS and control.

### 3.3 Osmolytes accumulation

**Fig 6:**
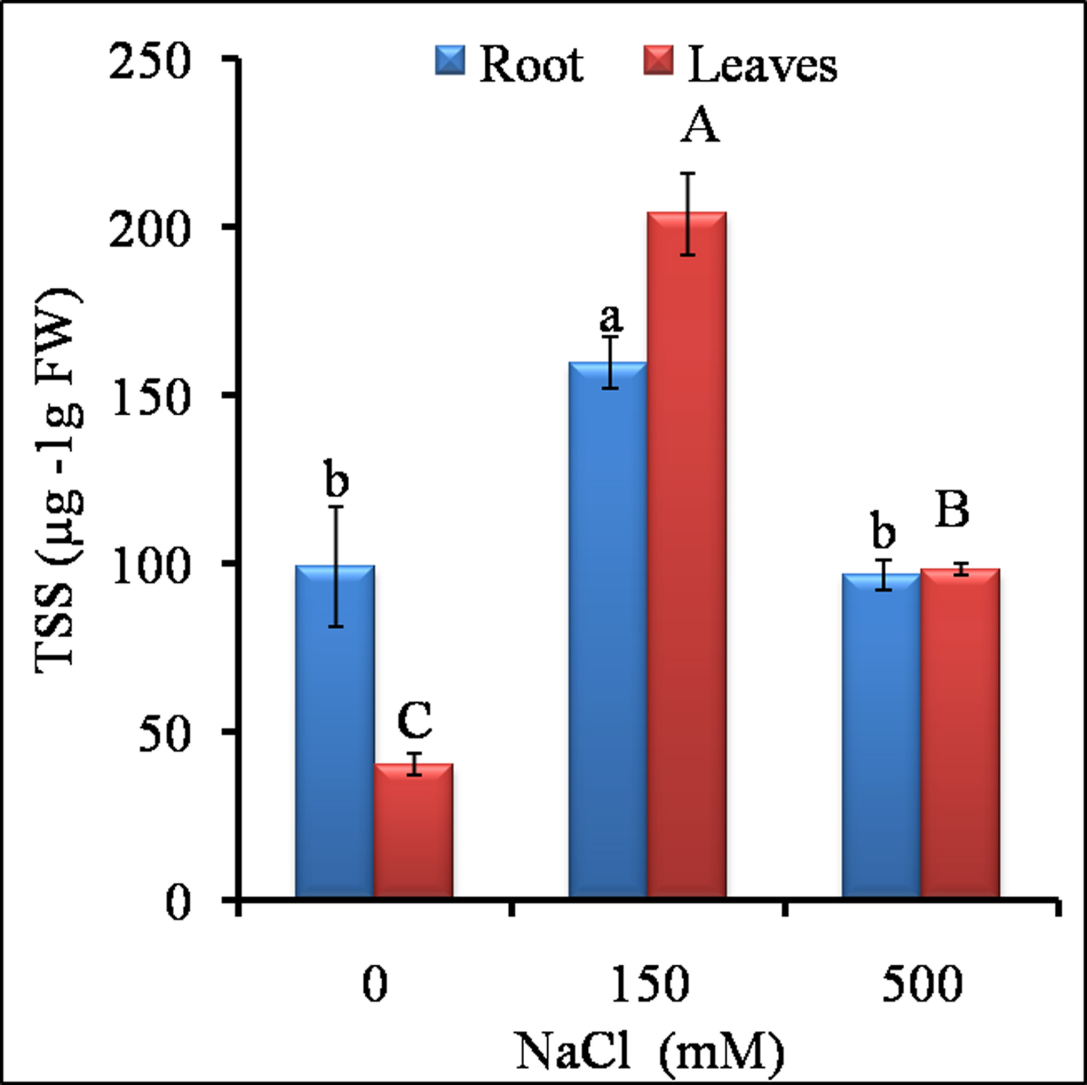
Effect of different NaCl treatments on TSS content of roots and leaves of *S*. *portulacastrum*. Different letters over bar with same color indicates significant difference in treatment according to Duncan's multiple range test DMRT *p*≤ 0.05).

**Fig 7:**
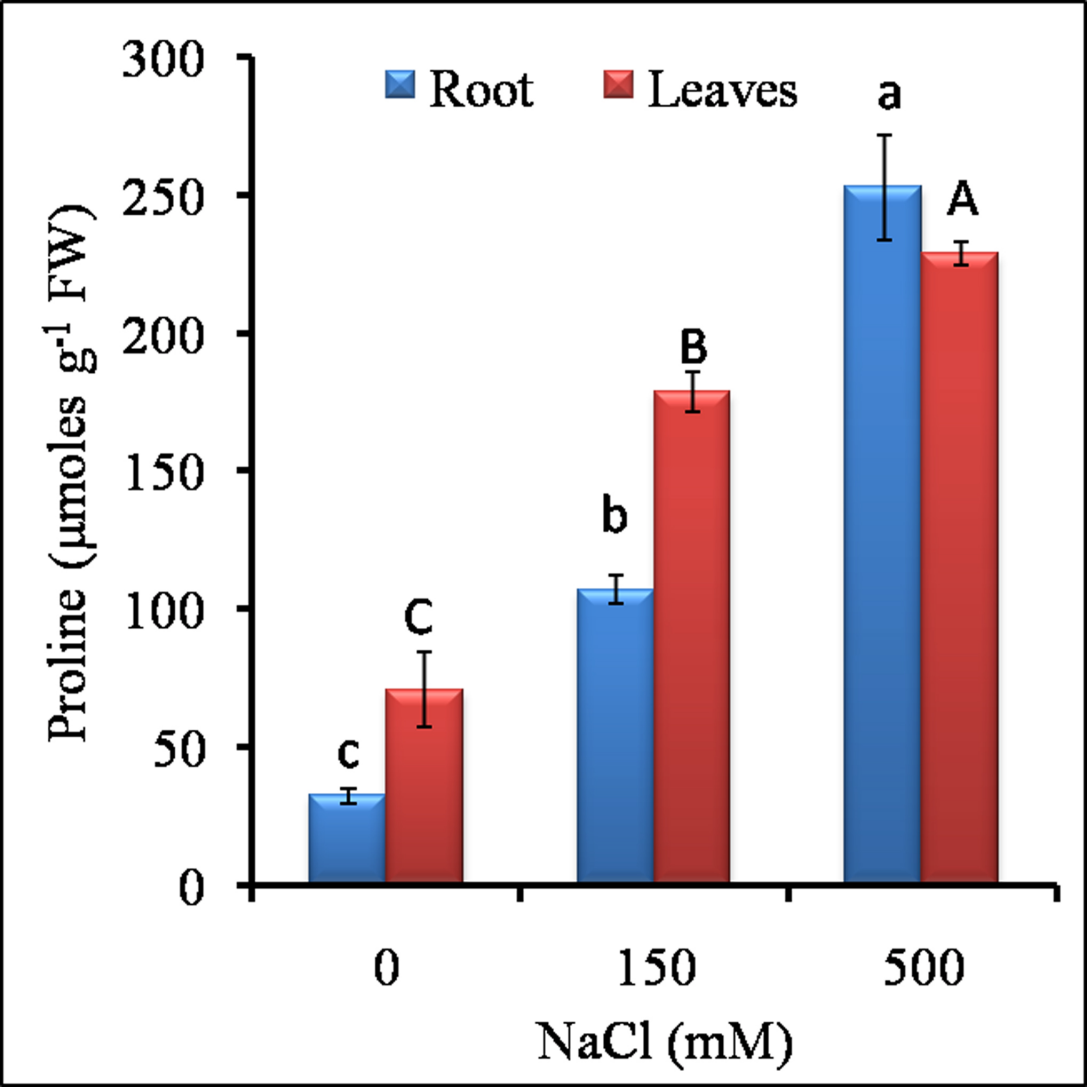
Effect of different NaCl treatments on proline content in roots and leaves of *S*. *portulacastrum*. All the values are means ±S.E. (n = 3). Different letters over bar with same color indicates significant difference in treatment according to Duncan's multiple range test (DMRT p≤ 0.05).

As compared to control, the TSS content in both the root and leaves was increased in LS treatment (Fig 6). In root, TSS was 1.6 fold higher in LS as compared to both control and HS while in leaves, TSS accumulation was 5 fold and 2 fold higher in LS as compared to control and HS respectively (Fig 6). The proline content in roots and leaves was increased in both LS and HS. In roots, proline content was 3.3 and 7.8 fold in LS and HS over control. In leaves, the proline content was about 2.5 and 7.8 fold high as compared to control (Fig 7).

### 3.4 Cation accumulation

**Fig 8:**
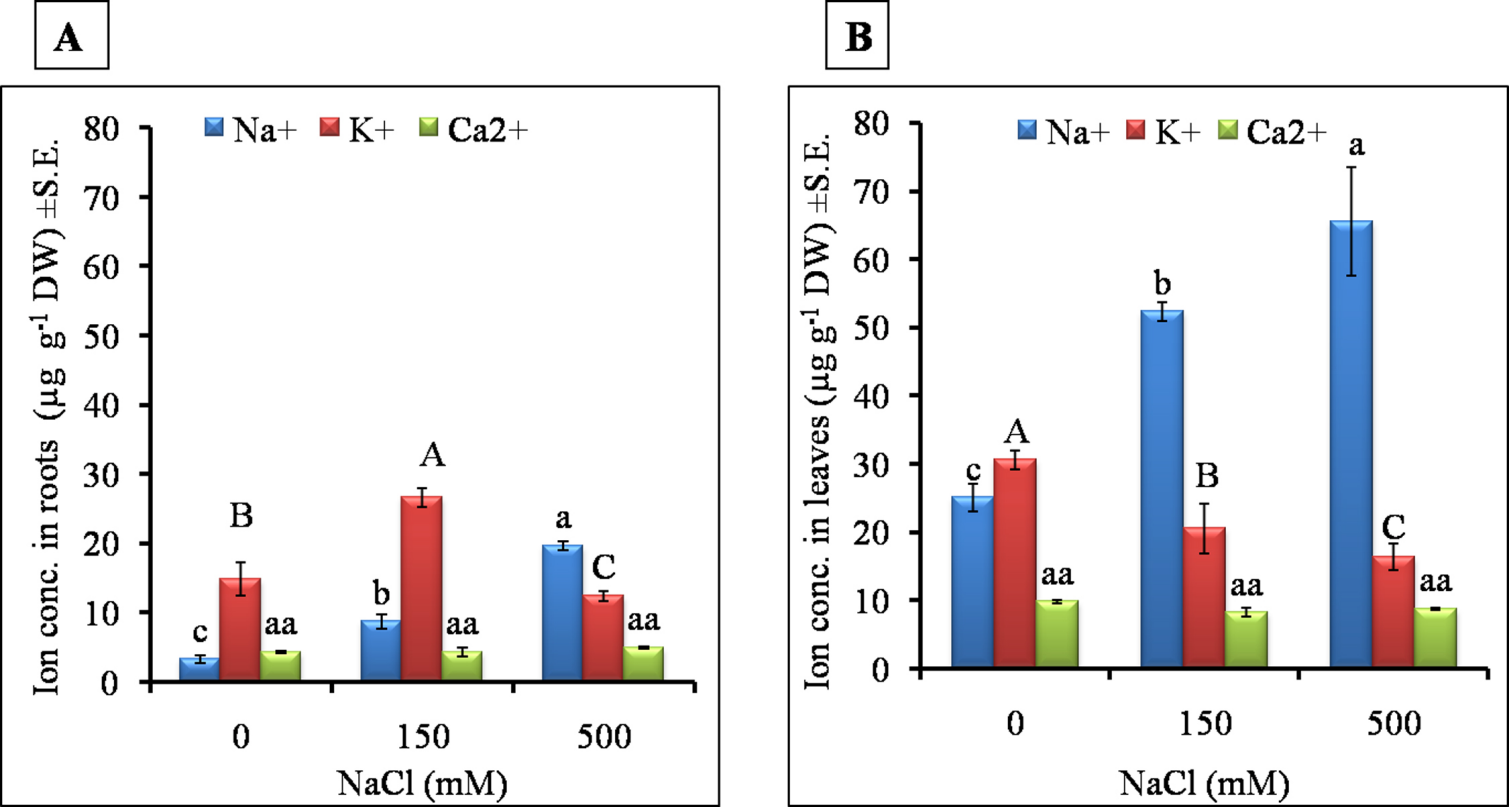
Effect of different NaCl) treatments on sodium, potassium and calcium ion content A) in roots, B) in leaves of *S*. *portulacastrum*. Different letters over bar with same color indicates significant difference in treatment according to Duncan's multiple range test DMRT *p*≤ 0.05).

After 28 days of salt treatment, cation content (Na^+^, K^+^ and Ca^2+^) in roots and leaves was measured. Salt stress, in general, induced high sodium (Na^+^) accumulation in roots and leaves (Fig 8). Na^+^ content in roots was increased by 2 and 6 fold in LS (8.67 μg.g^-1^ DW) and HS (19.61 μg.g^-1^ DW) respectively, as compared to control (3.26 μg.g^-1^ DW) (Fig 8A). In leaf, Na^+^ content was 2.4 and 3.2 fold higher in LS (52.27 μg.g^-1^ DW) and HS (65.50 μg.g^-1^ DW) respectively. A three-fold higher sodium accumulation was observed in HS in leaves than in the root (Fig 8B).

Potassium (K^+^) ion showed differential accumulation in roots and leaves. In roots, K^+^ content increased significantly in LS (26.58 μg.g^-1^ DW) as compared to the control (14.80 μg.g^-1^ DW) and HS (12.37 μg.g^-1^ DW) (Fig 8A). Leaf K^+^ content was decreased with increment in salt concentration, and was significantly lower in the salt treated plants as compared to control plants. In control leaves, the highest potassium accumulation (30.55 μg.g^-1^ DW) was observed than LS (20.49 μg.g^-1^ DW) and HS (16.32 μg.g^-1^ DW) (Fig 8B). Calcium (Ca^2+^) content was unaffected in root and leaves under both the treatments, and was similar to control (Fig 8A and 8B).

#### 3.4.1 Temporal and spatial accumulation of sodium and potassium

**Fig 9:**
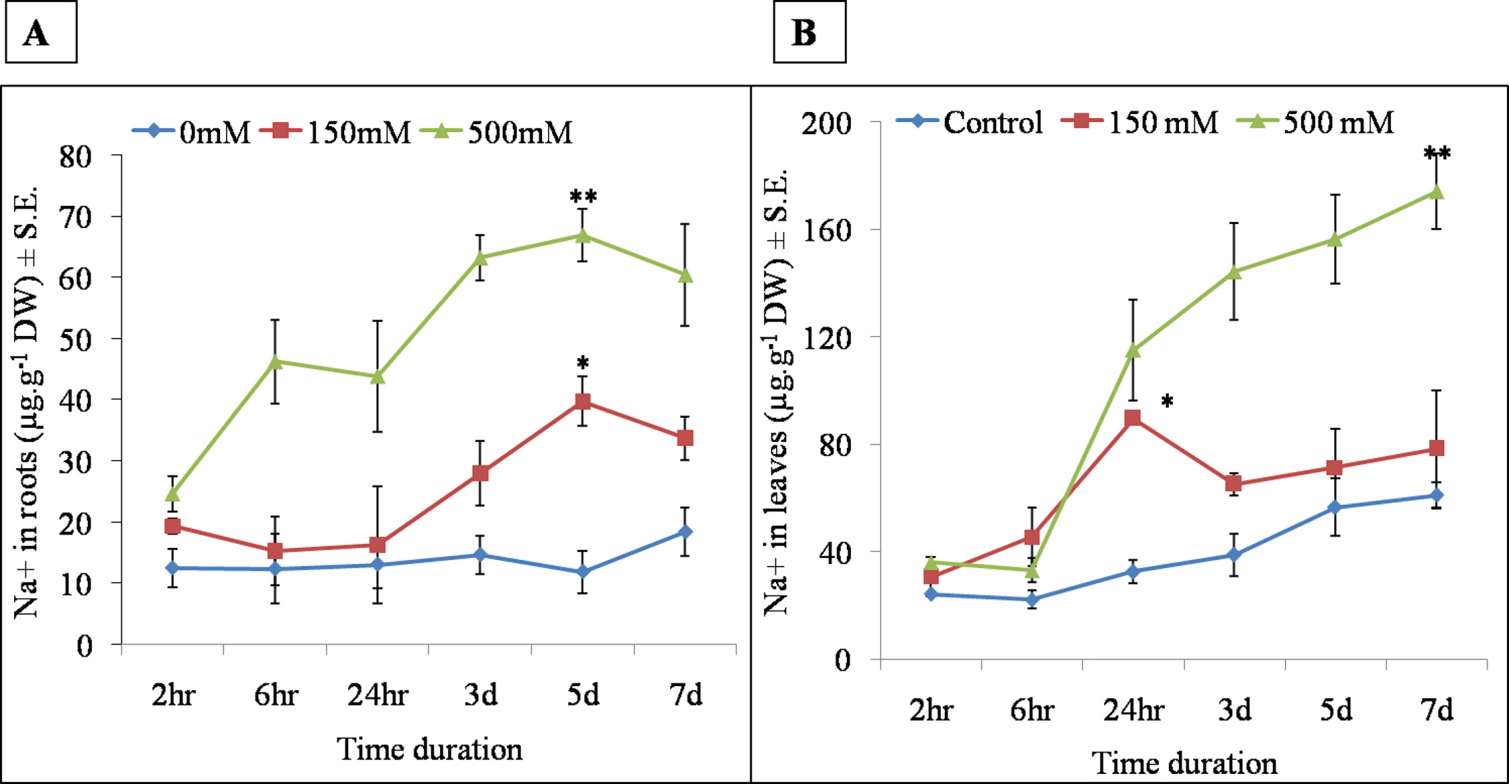
Time dependant accumulation of A) sodium in roots B) sodium in leaves at 2hr, 6hr, 24 hr, 3d, 5d and 7d in *S*. *portulacastrum* under 0, 150 and 500 mM NaCl treatment.

**Fig 10:**
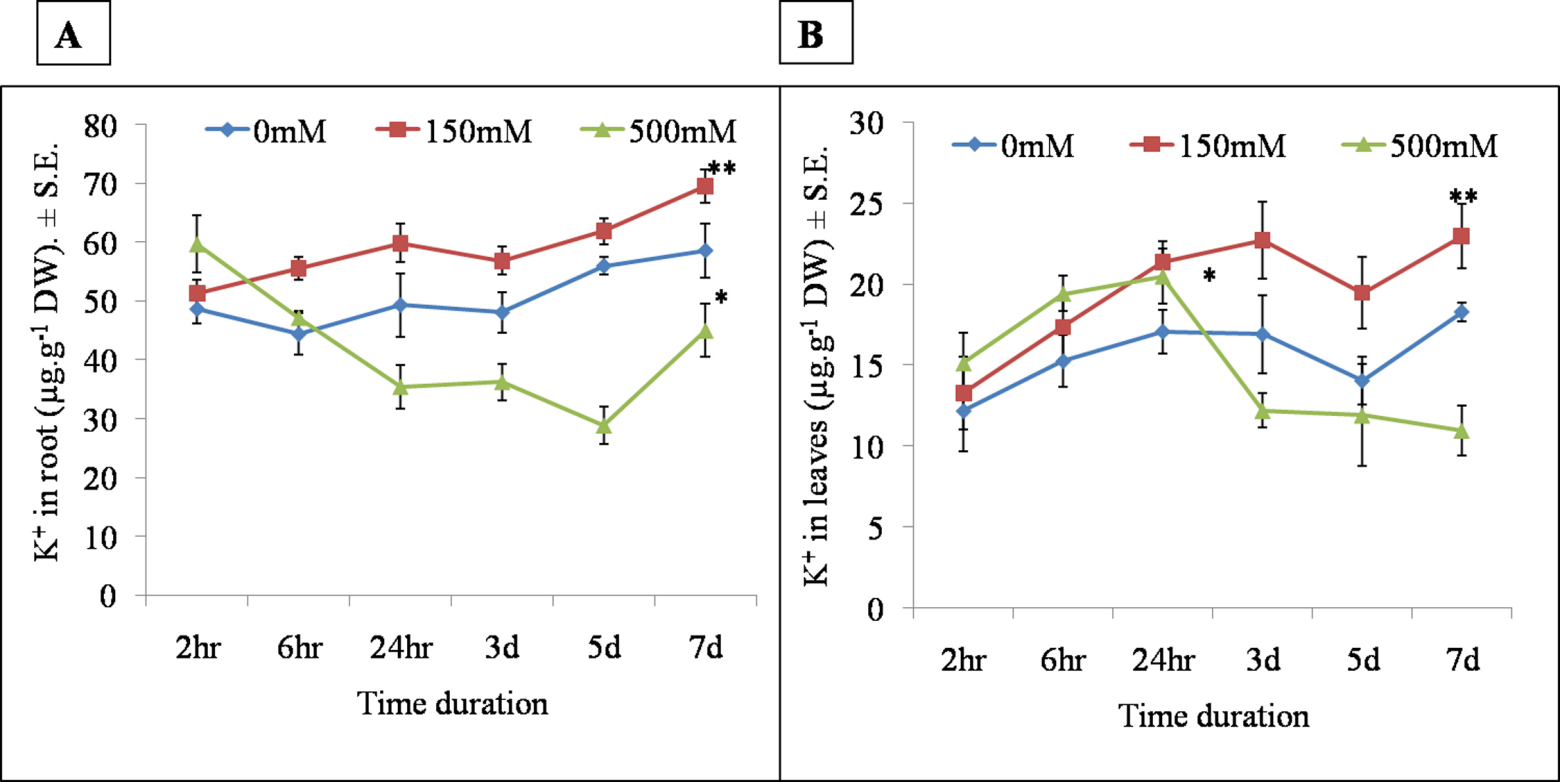
Time dependant accumulation of C) potasssium in roots D) potassium in leaves at 2hr, 6hr, 24 hr, 3d, 5d and 7d in *Sesuvium portulacastrum* under 0, 150 and 500 mM NaCl treatment. All values are means of three independent biological replicates. The significance of mean difference (P<0.05) was evaluated on the basis of student T-test and marked with asterisk (*).

To investigate the temporal and spatial cation accumulation under LS and HS treatments, we studied accumulation pattern of Na^+^, K^+^ in root and leaf over different time points (2hr, 6hr, 24 hr, 3d, 5d, 7d; Figs 9 and 10). Root Na^+^ content increased up to 5d but decreased later by the 7^th^ day in both LS and HS. At each time point, HS showed more than two fold higher sodium content as compared to LS (Fig 9A). Shoot Na^+^ showed significantly higher accumulation in response to stress treatment duration (Fig 9B). After 6hr of stress, sodium content was significantly increased in both LS and HS. In HS, the sodium content was increased gradually but in LS, increase in accumulation was only observed at 24hr salt treatment. After 2 hr, two-fold higher sodium content was recorded in HS as compared to LS in leaves (Fig 9B).

In roots, significantly high potassium content was observed at all the time points in LS as compared to HS (Fig 10A). In LS, potassium content of roots was gradually increased with time, while in HS it was decreased up to 5^th^ day with a slight increase by 7^th^ day (Fig 10A). In leaves, the potassium content showed the same trend but was almost two fold lower as compared to root (Fig 10B).

### 3.5 Hydrogen peroxide (H_2_O_2_) content

**Fig 11:**
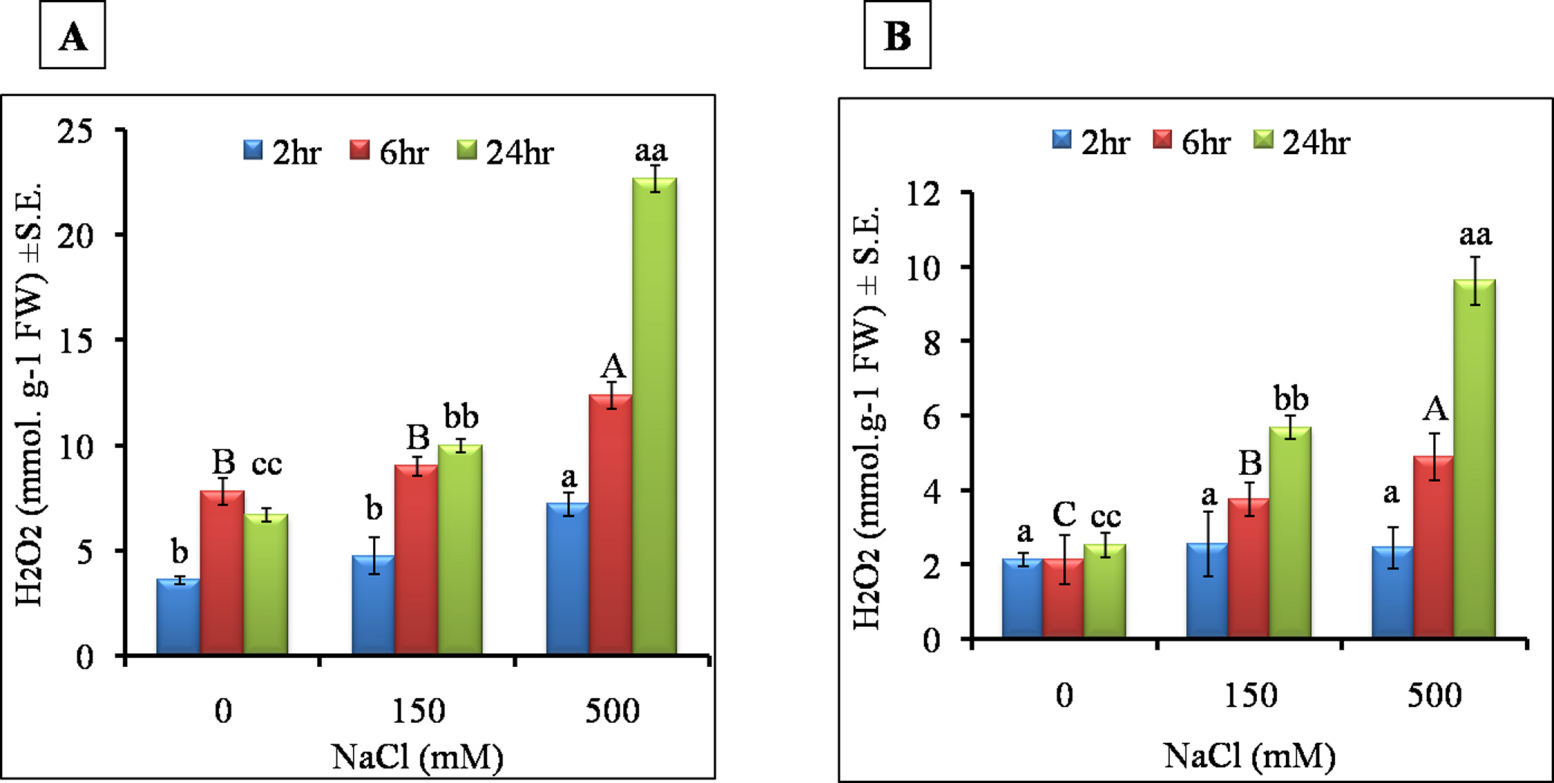
Effect of different NaCl treatments on hydrogen peroxide in A) roots and B) leaves of *S*. *portulacastrum* at different time points. All the values are means ±S.E. (n = 3) Different letters over bar with same color indicates significant difference in treatment according to Duncan's multiple range test (DMRT *p*≤ 0.05).

In general, roots accumulated higher H_2_O_2_ than leaves (Fig 11). The increase in H_2_O_2_ content was gradual and significant under HS during 2 hr to 24 hr salt treatment (from 2 to 3.4 fold) while in LS, the increase was marginal (from 1.3 to 1.5 fold), as compared to control (Fig 11A). In leaves, the HS induced higher H_2_O_2_ ranging from 1.14 to 3.8 fold during 2hr to 24hr of treatment while in LS, the increase was ranged from 1.2 to 2.24 fold during 2hr to 24hr of treatment, respectively (Fig 11B).

### 3.6 Antioxidant enzyme activity

**Fig 12:**
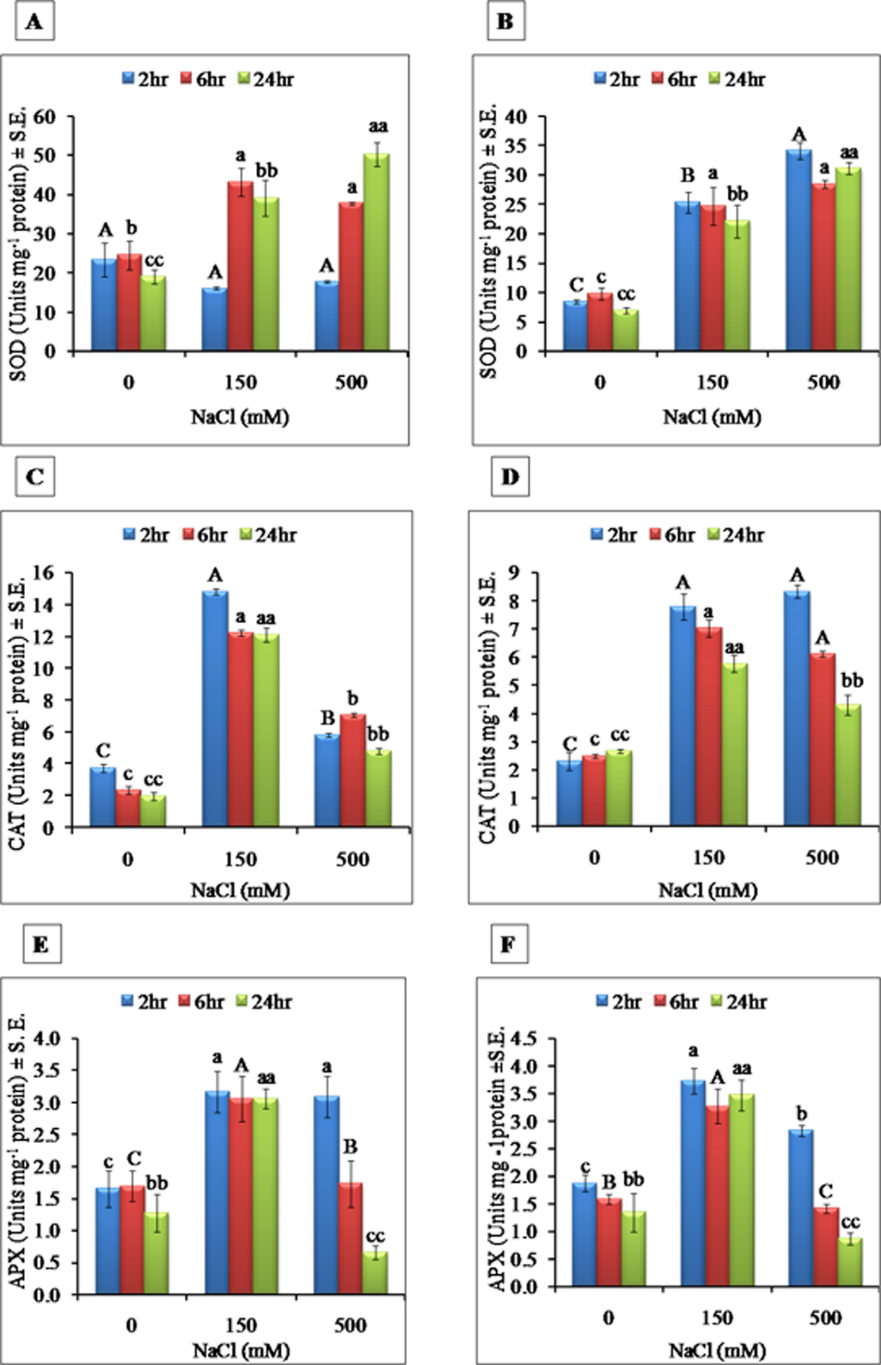
Effect of different NaCl treatments on superoxide dismutase enzyme activity in **A)** roots and **B)** leaves of *S*. *portulacastrum* at different time points. All the values are means ±S.E. (n = 3) Different letters over bar with same color indicates significant difference in treatment according to Duncan's multiple range test (DMRT p≤ 0.05). Effect of different NaCl treatments on Catalase activity in **C)** roots and **D)** leaves of S. portulacastrum at different time points. All the values are means ±S.E. (n = 3) Different letters over bar with same color indicates significant difference in treatment according to Duncan's multiple range test (DMRT p≤ 0.05). Effect of different NaCl treatments on Ascorbate peroxidase activity in **E)** roots and **F)** leaves of *S*. *portulacastrum* at different time points. All the values are means ±S.E. (n = 3) Different letters over bar with same color indicates significant difference in treatment according to Duncan's multiple range test (DMRT p≤ 0.05).

In roots, Superoxide dismutase (SOD) activity was significantly higher as compared to CAT and APX under control condition. Under both salt treatments, SOD activity showed gradual increase with time as compared to their respective controls. In HS, SOD activity was 0.7 to 2.64 and in LS, 0.6 to 2.03 fold high as compared to control from 2hr to 24hr respectively (Fig 12A). In leaves, the SOD activity was increased by 1.3 to 1.4 fold under HS as compared to control, and in LS, SOD activity increased by 2.99 to 3.2 fold from 2hr to 24hr respectively (Fig 12B).

In roots, catalase (CAT) activity was significantly increased at 2hr under both HS and LS (Fig 12C and 12D). In HS, CAT was increased from 1.56 fold, 3.0 and 2.44 fold after 2hr, 6hr and 24hr of NaCl treatment respectively, as compared to control. In LS, higher activity was observed from 3.97 to 5.2 and 6.15 fold after 2hr, 6hr and 24hr of salt treatment respectively (Fig 12C). In shoot, HS induced slightly higher CAT activity as compared to control while in LS, CAT activity was increased by 3.4 fold than control at 2 hr and later the activity was decreased by 2.8 to 2.1 fold at 6hr and 24hr (Fig 12D).

In roots, Ascorbate peroxidase activity (APX) was increased significantly by 1.9 fold in both LS and HS in 2hr treatment as compared to control but the activity was decreased at other time points in HS, whereas in LS, constant increase was observed (from 1.9 to 2.4 fold) as compared to control (Fig 12E). In leaves, APX activity showed a similar trend like root. In HS, the decrease was gradual (from 1.5 to 0.6 fold) whereas in LS, increased activity was observed with time (from 1.98 to 2.6 fold) (Fig 12F).

### 3.7 Expression analysis of transporters

**Fig 13:**
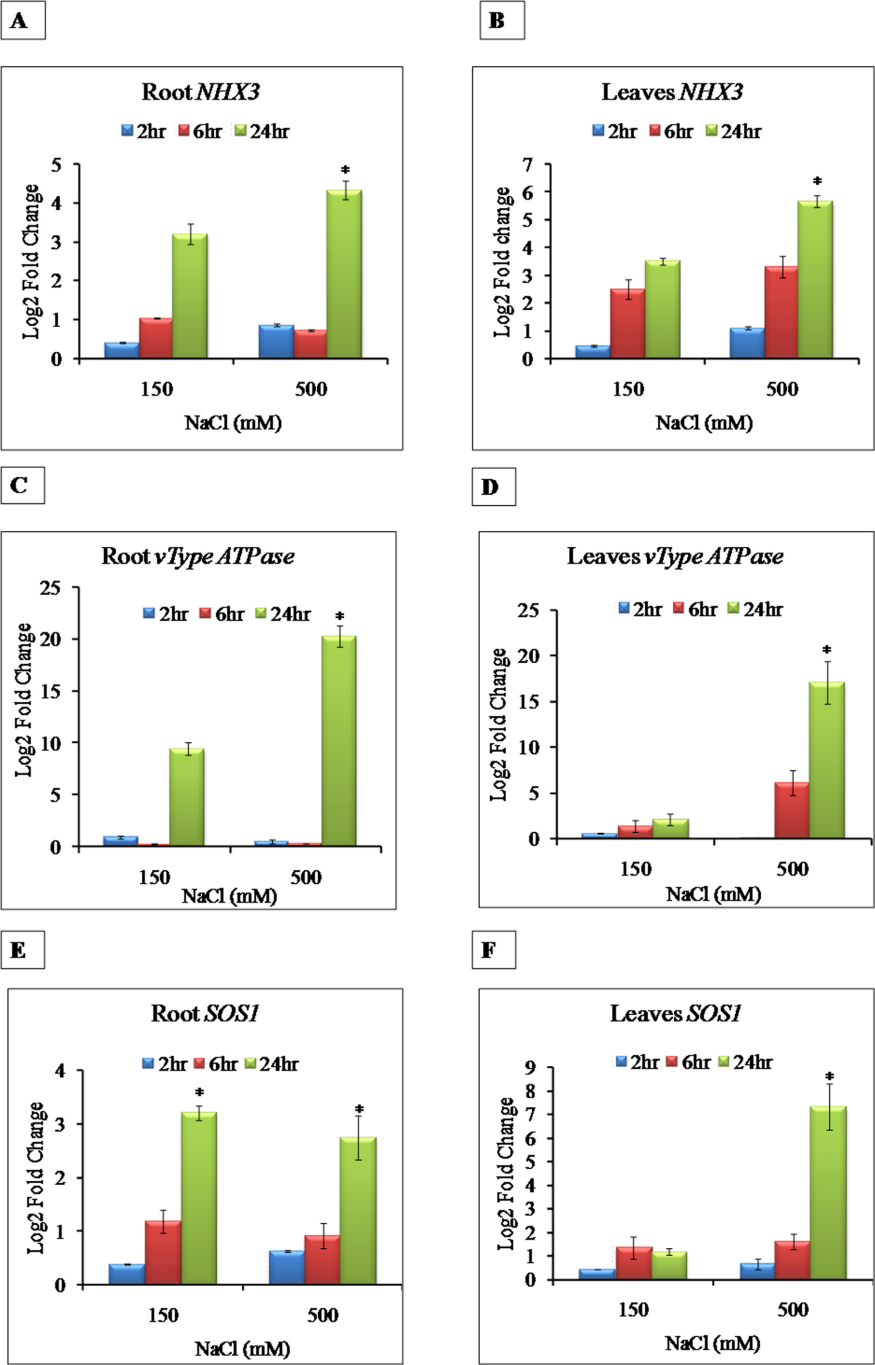
Expression profiling of three sodium transporters under different salt treatments and time points **A)** root NHX3, **B)** leaf NHX3 gene in *S*. *portulacastrum*. All values are means of three independent biological replicates. The significance of mean difference (P<0.05) was evaluated on the basis of student T-test and marked with asterisk (*). Expression profiling of three sodium transporters under different salt treatments and time points **C)** Root v-Type ATPase **D)** Leaves v-Type ATPase *S*. *portulacastrum*. All values are means of three independent biological replicates. The significance of mean difference (P<0.05) was evaluated on the basis of student T-test and marked with asterisk (*). Expression profiling of three sodium transporters under different salt treatments and time points **E)** Root *SOS1*, **F)** Leaves *SOS1S*. *portulacastrum*. All values are means of three independent biological replicates. The significance of mean difference (P<0.05) was evaluated on the basis of student T-test and marked with asterisk (*).

The results on the time dependant ion accumulation during the early time points (2–24 hrs) indicated modulated expression of ion transporters in *Sesuvium* (Fig 13). Hence, to understand role of ion transporters in differential accumulation of sodium and potassium, we studied expression of *NHX3*, *v-Type ATPase* and *SOS1* transporter genes at 2, 6 and 24hr NaCl treatment. In general, all the transporter genes were highly up regulated at 24hr in both the LS and HS treatments, in both roots and leaves (Fig 13).

#### 3.7.1 Expression pattern of *NHX3* under salt stress

Expression of *NHX3* was concentration and time dependant in the roots. In both the treatments, *NHX3* expression showed a gradual increase over control. At 24hr salt treatment, the *NHX3* expression was the highest. The *NHX3* expression in roots was 3.2 fold higher in LS while it was 4.3 fold higher in HS as compared to control (Fig 13A). In leaves, the expression of *NHX3* was induced at 6 hr of salt treatment. In LS, *NHX3* showed highest expression (22.54 fold) which dropped down by 24 hr of salt treatment compared to 2.0 fold of *NHX3* expression in HS (Fig 13B).

#### 3.7.2 Expression pattern of *v-Type ATPase* under salt stress

The *v-Type ATPase* was significantly up regulated in roots as compared to leaves (Fig 13C and 13D). In roots, after 24hr of salt treatment, *vType ATPase* was significantly up regulated as compared to 2hr and 6hr. Higher *vType ATPase* induction (20.29 fold) was observed in HS compared to LS (9.46 fold) (Fig 13C). In leaves, the *vType ATPase*induction was observed at 6hr of salt treatment. In LS, the expression was increased from 1.39 fold(6hr) to 2.1 fold(24hr) whereas in HS, the increase was significant from 6.17 fold (6hr) to 17.06 fold (24hr) (Fig 13D).

#### 3.7.3 Expression pattern of *SOS1* under salt stress

In roots, there was no significant change in expression of *SOS1* gene at 6hr but induction of *SOS1* was significant at 24hr of salt treatment. In LS, expression was found to range from 1.19 (at 6hr) to 3.29 fold (at 24hr) and in HS, expression was increased from 0.91 fold to 2.74 fold (6hr to 24hr) (Fig 13E). Leaves showed contrasting expression of *SOS1* gene as compared to root. Similar to *NHX3* gene, in LS the expression of *SOS1* gene was induced at 6hr (11.2 fold) which was down regulated to 1.17 fold while in HS, increase was gradual from 0.43 to 1.62 to 7.3 fold (Fig 13F).

### 3.8 Correlation analysis

In salt-treated plants, a differential correlation pattern was observed between sodium accumulation and stress indicators (total chlorophyll, MDA, RWC and proline. The total chlorophyll (r^2^ = 0.82), MDA content (r^2^ = 0.77) and proline (r^2^ = 0.88) showed positive co-relation with sodium content in both the root and leaf tissues. The RWC showed a negative correlation with sodium accumulation only in HS (r^2^ = -0.73) while TSS showed poor correlation (r^2^ = 0.43).

### 3.9 Identification of differentially accumulated metabolites

**Fig 14:**
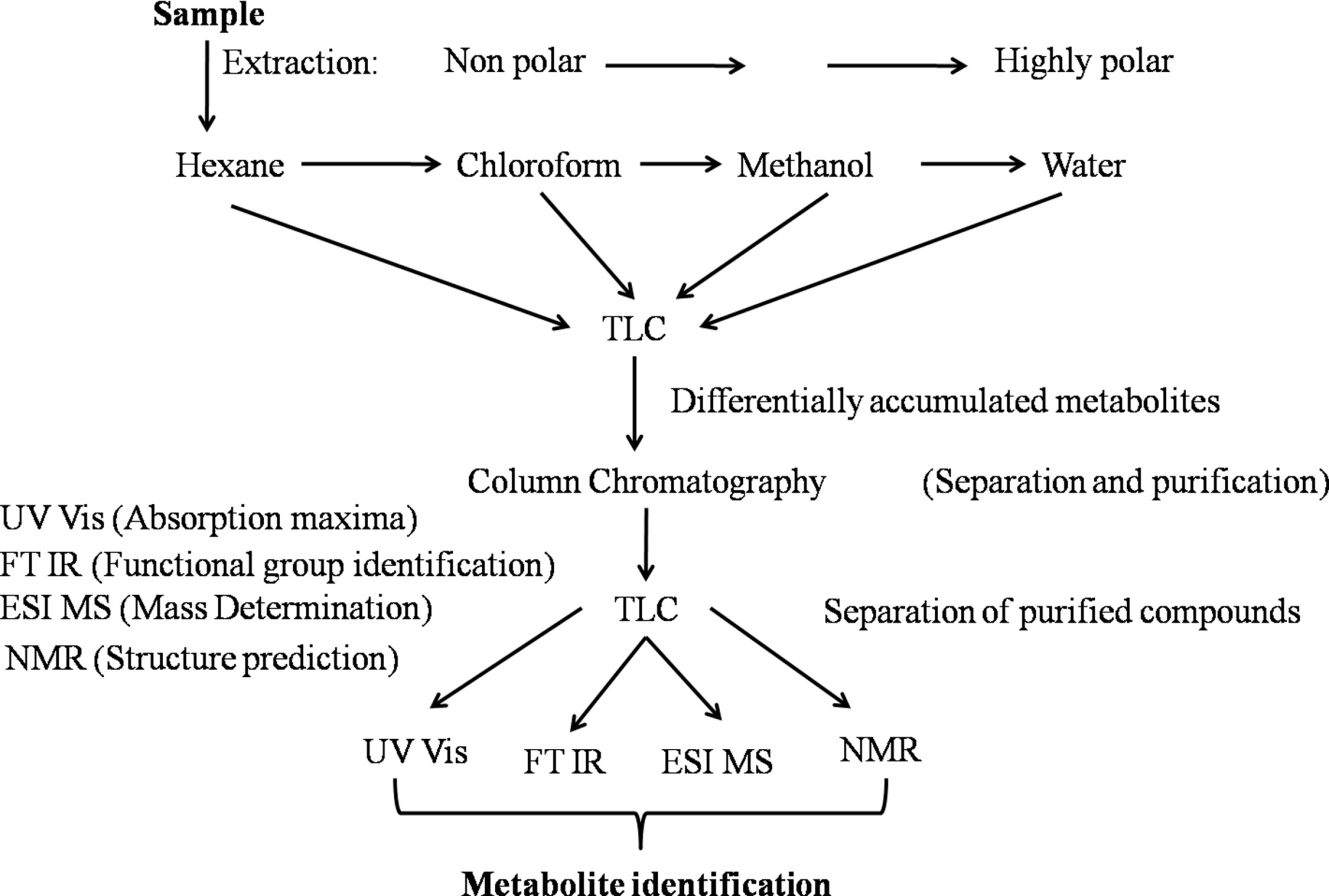
Workflow for extraction, separation, purification and identification of differentially accumulated metabolites from *S*. *portulacastrum* under different salt treatments.

**Fig 15:**
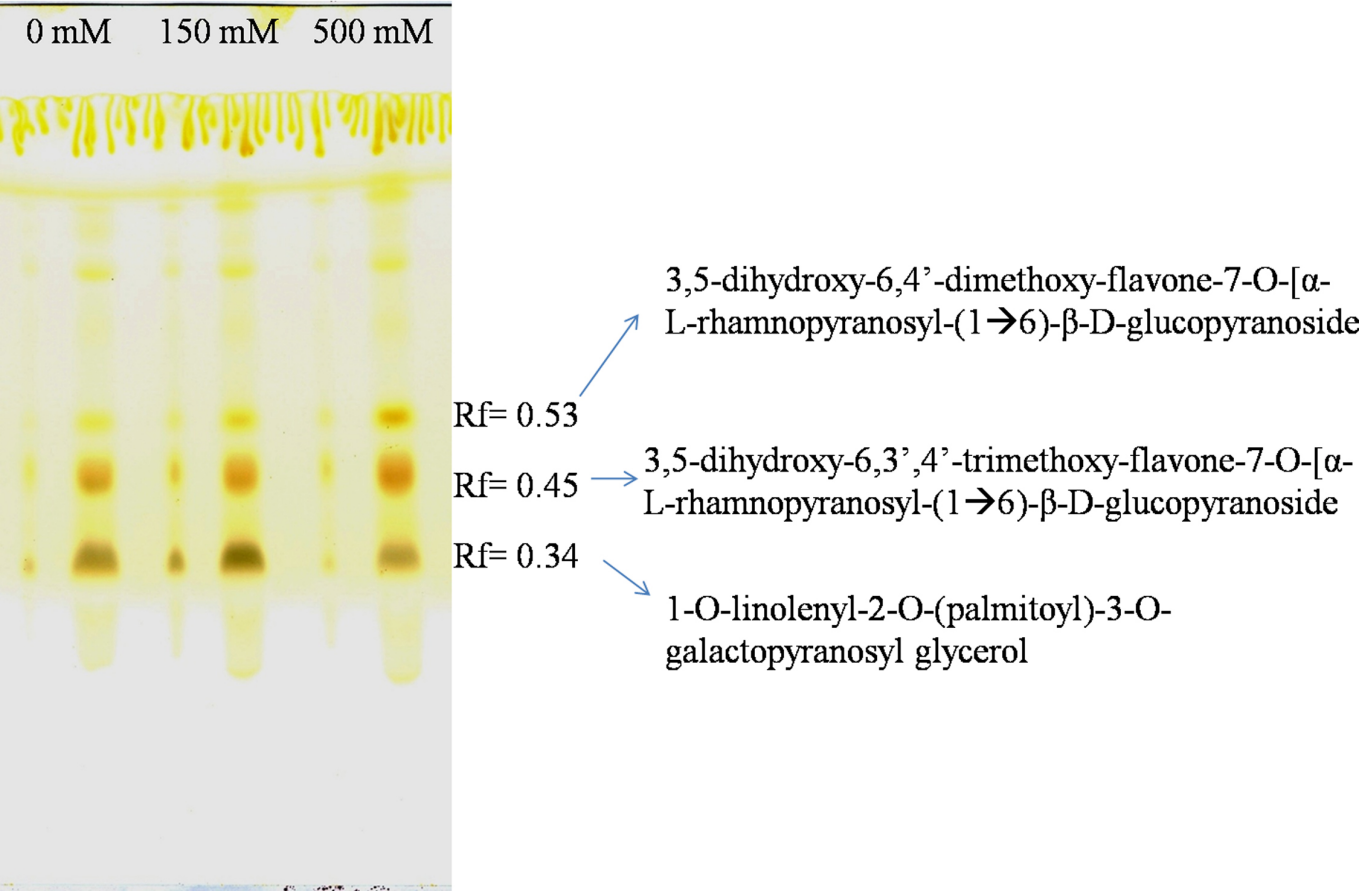
TLC of methanolic extract of *Sesuvium portulacastrum*. Sample was loaded in both spot and band form) from 0, 150 and 500 mM NaCl treatment.

Since sodium accumulation was high in leaves, we proposed to study the metabolite isolation and identification in the leaf tissue. The lyophilized leaf samples were successively extracted with different solvent systems: hexane, chloroform, methanol and water and the extracts were run on TLC. The workflow of extraction, separation, purification and identification of differentially accumulated metabolites from *S*. *portulacastrum* under different salt treatments is given in Fig 14. Of all the extracts that were run on the TLC only methanolic extract showed three distinct and differential intensity bands (R_f_ values of 0.53, 0.45 and 0.34) under salt stress treatment (Fig 15).The individual bands were further isolated by column chromatography and characterization by using different analytical methods such as UV vis spectroscopy, FT IR, ^1^H NMR, ESI-MS. Compounds at R_f_ values of 0.53 and 0.45 were eluted in 80:20 and 70:30 methanol: water fraction while that at R_f_ 0.34 was eluted with methanol: water (60:40).

**Compound I** (80 mg/100 g) was isolated as a yellow amorphous powder. An intensification of yellow color of the separated spot (R_f_ 0.53) on TLC plate when sprayed with H_2_SO_4_ indicated its flavonoid nature (Fig 15).

The flavanoid nature of compound was further confirmed by its UV absorption maxima at 271 and 341 nm. IR absorption at 3400, 1660, 1590 and 1459 cm^-1^ suggested the molecule to have hydroxyl groups, α-β unsaturated carbonyl group and with aromatic nature.

The spectrum obtained by ESI-MS analysis showed a prominent sodiated molecular ion at m/z 661 [M^+^Na]^+^ in the mass spectra.

The ^1^H NMR spectrum showed doublets at δ 6.86 and 8.15 (each 2 H, J = 8.5 Hz) indicating the presence of para-di-substituted aromatic ring which represented an A2B2 system in the ring B, and this could be attributed to the signals from 2’/6’ and 3’/5’protons respectively. A down field singlet at δ 6.7 was assigned to C-8 protons. Singlet signals at δ 3.98 and 3.88 indicated the presence of two methoxyl groups in the molecule. A doublet at δ 5.12 (H-1”, d 7.5 Hz) was assigned to the anomeric proton of inner glucose with a β-D-glucosyl linkage. The terminal sugar unit with an anomeric proton at δ 4.57 (broad singlet) was identified as a rhamnose moiety having α-L configuration. The presence of rhamnose was also further confirmed from a characteristic methyl doublet at δ 1.20 (d 6Hz, H3-6”‘). Other sugar protons gave unresolved signals between δ 3.3–3.79.

Based on the above spectral data and comparison with literature reports the compound was tentatively identified as 3, 5-dihydroxy-6, 4’-dimethoxy-flavone-7-O-[α-L-rhamnopyranosyl-(1→6)-β-D-glucopyranoside].

**Compound II** (80 mg/100g) was resolved on TLC at R_f_ 0.45 and this showed an enhanced yellow color when sprayed with H_2_SO_4_ indicating its flavonoid nature (Fig 15).

The flavanoid nature of compound was confirmed by its UV absorption maxima at 285 and 339 nm. IR absorption at 3400, 1660, 1590 and 1459 cm^-1^further suggested the molecule to have hydroxyl groups, α-β unsaturated carbonyl group and an aromatic nature.

The spectrum obtained by ESI-MS analysis showed a prominent sodiated molecular ion at m/z 691 [M^+^Na]^+^ and a sodiated aglycone at m/z 383 in the mass spectra.

Proton signals in the ^1^H NMR spectrum at δ 6.8, 7.35 and 8.1 were assigned to H-5’, H-6’ and H-2’in the aromatic B ring. Singlet signals at δ 3.91 and 4.08 and 4.15 indicated the presence of three methoxyl groups in the molecule. A doublet at δ 5.40 (H-1”, d 7.5 Hz) was assigned to the anomeric proton of inner glucose with a β-D-glucosyl linkage. An anomeric proton at δ 4.50 (broad singlet) was identified as terminal rhamnose moiety having α-L configuration further confirmed by a characteristic methyl doublet at δ 1.35 (d 6Hz, H3-6”‘). Unresolved signals between δ 3.2–3.8 were assigned to other sugar protons. Based on the above spectral data and comparison with literature reports, the compound was tentatively identified as 3, 5-dihydroxy-6, 3’, 4’-trimethoxy-flavone-7-O-[α-L-rhamnopyranosyl-(1→6)-β-D-glucopyranoside].

**Compound III** This compound (66 mg/100g) was resolved on TLC at R_f_ of 0.34 (Fig 15). The UV Visible and FT IR analysis of this compound showed a broad absorption band at 3382 cm^-1^ indicating the presence of OH group in the molecule (Fig 11). It showed UV absorption maxima at 273 nm. An IR signal at 1740 cm^-1^ was assigned to ester carbonyl groups while that at 2927 and 2856 cm^-1^ was inferred to be due to aliphatic CH stretching.

The spectrum obtained by ESI-MS analysis revealed a sodiated molecular ion at m/z 776 [M^+^Na]^+^ indicating a molecular formula of C_43_H_76_O_10_Na.

The ^1^H NMR spectrum showed signals at δ 4.15 and 4.28 that corresponded to C-1 protons of glycerol substituted by an O-acyl group. A multiplet at δ 5.28 was assigned to C-2 while doublets at δ 3.70 and 3.80 were assigned to C-3 protons of glycerol substituted by β-galactose. Two terminal methyl groups of fatty acid acyl chains were assigned to signals at δ 0.85 (3H, t, J = 6.9 Hz) and 0.95 (3H, t, J = 7.5 Hz). A broad signal (δ 1.25–1.35) was assigned to methylene protons of the two aliphatic chains of the two acyl groups while multiplets centered at δ 1.65, 2.0 and 2.25 assigned to methylene groups linked α, β to ester functional groups. A signal at 2.75 (4H, t, 5.7 Hz) could be attributed to methylene group of unsaturated fatty acyl group such as linolenic acid. Olefinic methane proton signals were present as a cluster at δ 5.25–5.42. Signals in the region δ 3.6–4.0 were attributed to sugar protons. Based on the above spectral data and comparison with literature reports, the compound was tentatively identified as 1-O-linolenyl-2-O-(palmitoyl)-3-O-galactopyranosyl glycerol.

The results showed an increased accumulation of 3,5-dihydroxy-6,4’-dimethoxy-flavone-7-O-[α-L-rhamnopyranosyl-(1→6)-β-D-glucopyranoside], by 1.3 and 1.8 fold and of 3,5-dihydroxy-6,3’,4’-trimethoxy-flavone-7-O-[α-L-rhamnopyranosyl-(1→6)-β-D-glucopyranoside] by 1.15 and 1.16 fold in LS and HS respectively.

## 4. Discussion

Soil salinity imposes deleterious effects on all the plant species including halophytes; however halophytes possess high salt tolerance by virtue of adaptive mechanisms which include ion homeostasis, compatible solutes, antioxidant machinery and biochemical defense [51]. *Sesuvium portulacastrum* is a perennial halophyte with a fast growing pattern in salt marshes. Although salt responsive physiological effects were documented previously in this plant [33, 52], insight into the salinity induced ionic imbalances and biochemical and metabolic alterations is essential to further understand salt adaptation mechanism. In the present work, we studied differential root and leaf growth responses, temporal, spatial cation accumulation and possible involvement of chemical compounds in *Sesuvium* to strengthen defense of *Sesuvium* against high salinity.

Most halophytes manage their growth through the regulation of water content and osmotic adjustment under growth limiting conditions [53]. It has been suggested that osmotic stress results in immediate osmotic adjustment in the root elongating zone contributing to the maintenance of turgor pressure and root growth [54]. Our results showed that the *Sesuvium* plants performed better without much growth reduction under low salt treatment conditions (LS, 150 mM NaCl). The root length of LS plants was increased significantly which may be the result of reduced osmotic potential. Increase in root to shoot ratio is related to osmotic effect rather than a salt-specific effect [55]. In halophytes like, *Haloxylon ammodendron*, *Suaeda physophora* and *Salsola nitraria*, increase in root length was observed under low concentration of NaCl [56]. Abideen et al. [57] also observed that medium level of salinity (100 mM) could increase leaf area, shoot height and root/shoot ratio in *Phragmites karka*. Differential root and shoot growth patterns have also been observed in other halophytes. In *Pennisetum alopecuroides* increased root length was observed at 50 mM NaCl [58] while, in *Porteresia coarctata*, root length was significantly increased in 400 mM NaCl treatment [59]. In our study, the reduced growth under HS was associated with reduction in relative water content and fresh weight. This reduced growth could save energy, which can be reallocated towards synthesis of osmolytes and antioxidants, which protects plants from excessive ROS load and further damage [60]. High salinity also affected leaf area and subsequently photosynthetic pigments. In *Prosopis strombulifera* chlorophyll pigments were decreased by 1.7 fold as compared to control under salt stress [61] while *Mesembryanthemum* did not show any change in chlorophyll content under stress [62]. Our results however, showed significant increase in chlorophyll content under both salt treatments (Fig 3) which suggests probable requirement of salt for optimal growth in *Sesuvium*. Such a relationship between growth, photosynthetic activity and salt tolerance has been shown in some halophyte plants [35].

The oxidative stress indicators like MDA content, chlorophyll pigments, proline, RWC and TSS are considered important for studying salt induced damage [13, 39] Our results showed a positive correlation between MDA, proline and chlorophyll with sodium content indicating that salt treatments impose oxidative stress which increases proline and chlorophyll content suggesting maintenance of cellular and physiological homeostasis in halophytes. On the other hand, RWC and sodium accumulation showed negative correlation under HS. In *Aeluropus lagopoides*, a positive correlation was observed between MDA, proline and salt treatment and negative correlation between RWC and salt treatment [13]. The low RWC and high proline content may constitute one of the economical strategies of *Sesuvium* plants to cope with salt induced damage with minimum energy input [63].

Sodium is considered a cheap osmolyte which helps in the maintenance of cellular osmotic potential in halophytes [64]. In *Mesembryanthemum crystallinum*, Na^+^ ions are shown to be essential for the maintenance of cellular osmotic potential under high salt concentration [65]. In *Sesuvium*, our results showed a contrasting pattern of sodium and potassium ion accumulation in root and leaves under LS and HS treatment. Leaves accumulated almost three fold high sodium as compared to roots (Fig 8) which indicates an efficient sodium ion translocation from root to shoot. The temporal and spatial study of ion accumulation showed that after a 24 hr salt treatment, root sodium accumulation was lower than the leaf sodium content which showed a steady increase with treatment time. Many halophytic plants exclude toxic salt ions from root, for example *Porteresia coarctata* excludes salt ions from leaf salt hairs [59] but *Sesuvium* rapidly translocates sodium ion from root to leaves and sequesters in the vacuole [33]. This suggests that such rapid translocation may be necessary to keep the root zone free of toxic salt ions to enable normal root functioning. We also noted contrasting accumulation of potassium in both the root and leaf tissue of *Sesuvium*. Under LS, roots accumulated high potassium and leaves accumulated high sodium indicating that roots and leaves respond differently to salt treatments and differential requirement of cations for metabolism [66]. In halophytes like *Chenopodium quinoa*, higher leaf potassium content was noted with increase in NaCl treatment [67] while in *P*. *coarctata* leaf potassium content remained unaffected while root potassium decreased gradually with increase in salt treatment [59]. Shabala and Pottosin [68] suggested that cytosolic K^+^ content could be involved in regulating the transition of plant growth from normal metabolism to an altered or modulated state in the early hours after the stress exposure.

The ion transporters are involved in efflux of Na^+^ across plasma membrane and its compartmentalization into vacuole. The vacuolar (*NHX*) and plasma membrane Na^+^/H^+^ antiporters (*SOS1*) are two important components of plant salt tolerance mechanism which regulates Na^+^ efflux [69, 70]. In several halophytes, transporters have been shown to regulate sodium efflux [14]. However, in *Sesuvium*, there is no information available on the ion transport. In the present study, we have shown that the three transporters: *NHX3*, *v-Type ATPase* and *SOS1* were upregulated under salt treatment. Salt induced, expression of *NHX* gene triggers the activity of tonoplast based Na^+^/H^+^ antiporter [71], and up regulation of *NHX* alongwith *v-Type ATPase* and *SOS* genes may contribute to maintainthe electrochemical proton gradient across tonoplast [26]. In our study, we observed induction in expression of the tested transporter genes from 6 to 24hr of salt treatment. An early induction in the *NHX1* transcripts has been reported in *Atriplex gmelini*, *Mesembryanthemum crystallinum* and *Suaeda salsa*[72]. In *Mesembryanthemum* after 72hr of salt treatment, transcript level of *v-Type ATPase* was increased in leaves, but in roots, there was no significant change [23]. In contrast, we found high expression of *v-Type ATPase* in both roots as well as leaves in *Sesuvium*. Similarly, the *SOS1* gene of *Thellungiella halophila* showed increased expression in both roots and leaves under salt stress [19]. The increased activity of *NHX* and *SOS*genesin leaf leads to compartmentalization of sodium ions in leaf vacuoles [73] while increased expression of these genes in root may alleviate translocation of sodium from root to leaf under salt treatment [74].

Accumulation of compatible solutes is an important adaptive response towards salt tolerance as a counter mechanism to balance the osmotic potential of the toxic ion accumulation [14]. Proline and total soluble sugars act as osmolytes and protect plant from toxic effects of salt ions [5]. In our study, proline accumulation was higher in leaf followed by roots. In halophytes like *Triglochin maritima* L., free proline accounts for 10–20% of the shoot dry weight under salt stress conditions while maintaining low proline levels under non-saline conditions [75]. Pardo-Domènech et al. [76] reported high proline accumulation in two Mediterranean halophytes (*Plantago crassifolia* and *Inula crithmoides)* to contribute to osmotic balance when plants were treated with 450–600 mM NaCl. In our study, the root tissue showed higher TSS in LS and HS while in leaves it was 4.5 fold and 2 fold higher in LS as compared to control and HS (Fig 7). These results suggest that halophytes possess built-in mechanisms of synthesis of additional osmolytes to rapidly adapt to increasing salinity levels. We have shown that both the osmolytes (proline and TSS) were also positively correlated with increasing sodium content of the tissue. Positive correlation between osmolytes and sodium content has been shown in halophyte *Juncus* species [77], *Tamarix ramosissima* [78] and it is possible that mechanisms such as protection of membrane integrity and increased structural stability of ion transporters may also contribute to high salt tolerance [79].

Cellular antioxidant system through the activation of enzymatic or non enzymatic antioxidants is crucial for the maintenance of cellular redox homeostasis required for salt adaptation or tolerance response in different plants [80] including halophytes. Our results showed that reactive oxygen species (H_2_O_2_)was dramatically accumulated after 24 hr salt treatment in both roots and leaves under high salinity stress (Fig 11). The SOD enzyme is said to be the first line of defense against oxidative stress and catalyzes dismutaion of O_2_^-^ to H_2_O_2_ and O_2_[64]. The upregulation of APX and CAT under high salinity is one of the mechanisms of salt adaptation in halophytes [64]. In this study, SOD activity was increased significantly in both roots and leaves after 6 hr of salt stress in both LS and HS. However, CAT and APX activity was decreased gradually in HS as compared to LS. Failure in activating antioxidant enzyme activity, especially catalase, in HS could have resulted in sustained oxidative damage thereby leading to decreased root length and normal functioning of roots. The Leaf antioxidant enzyme activities also followed a similar trend. The CAT and APX managed to counterbalance excess ROS under LS but under HS antioxidants were severely affected which reflected in reduced fresh weight and shoot length. In halophytes, *Bruguiera gymnorrhiza* and *B*. *Parviflora* the SOD activity was increased significantly under salt stress [81]. In a halophyte, *Nitraria tangutorum* different concentrations of NaCl induced activity of SOD, CAT and APX enzymes [82]. The results point to the fact that the antioxidant enzyme system act as a defense arsenal for building an adaptive mechanism under high salinity in halophytes.

Halophytes show a wide chemical diversity and, the study of metabolomics at specific time under different environmental conditions has assumed significance [83]. For non-model plants, metabolic studies will necessarily require optimization. The workflow followed in this study was based on the chemical diversity of compounds that could be occurring in halophytes. The use of different solvents aided in the extraction of compounds having varied polarity. The TLC was used to identify differentially accumulated metabolites and different techniques were used to purify and identify those compounds. Stress induced induction of metabolites particularly flavonoids has been reported in different plant species [32]. It is interesting to note that the same conditions of salt stress which induce cellular damage through disruption of antioxidant machinery, also lead to an increase in flavonoids synthesis which suggests that they may contribute as a second line of ROS-scavenging system in plants exposed to salt stress conditions. In the present study, since sodium accumulation was high in leaves, we have isolated and characterized salt induced metabolites from leaves. Of all the extracts that were run on the TLC, only methanolic extract showed three distinct and differential intensity bands under salt stress treatment. These three bands were selected for further purification by column chromatography and characterization by using different analytical methods such as UV vis spectroscopy, FT IR, ^1^H NMR, ESI-MS. The flavonoid compounds were identified as 3,5-dihydroxy-6,4’-dimethoxy-flavone-7-O-[α-L-rhamnopyranosyl-(1→6)-β-D-glucopyranoside] and 3,5-dihydroxy-6,3’,4’-trimethoxy-flavone-7-O-[α-L-rhamnopyranosyl-(1→6)-β-D-glucopyranoside] while a glycolipid was identified as 1-O-linolenyl-2-O-(palmitoyl)-3-O-galactopyranosyl glycerol, an MGDG (monogalactosyldiacylglycerol). Increased accumulation of 3,5-dihydroxy-6,4’-dimethoxy-flavone-7-O-[α-L-rhamnopyranosyl-(1→6)-β-D-glucopyranoside], by 1.3 and 1.8 fold and of 3,5-dihydroxy-6,3’,4’-trimethoxy-flavone-7-O-[α-L-rhamnopyranosyl-(1→6)-β-D-glucopyranoside] by 1.15 and 1.16 fold was observed in LS and HS respectively.

Flavonoids are the largest group of polyphenols with a great ability to scavenge O_2_^·–^, OH·, H_2_O_2_ and ^1^O_2_ and have been shown to have an *in planta* antioxidant function[64, 84]. Based on total phenol content, halophytes including *Sesuvium* have been shown to have an antioxidant role in scavenging free radicals and are suggested to be a vital source of antioxidant phytochemicals[85,86]. In halophyte *Prosopis stombulifera*, Reginato et al. [61] showed that the pool of total phenols was composed mainly of flavan-3-ols in leaves, accumulating almost 40 to 45% under salt stress thus preventing plant from oxidative and ionic stress. High flavonoid content was also observed in halophytes exposed to different levels of salinity [87]. Stankovi et al. [88] screened inland halophytes for their antioxidant activity and found that concentration of flavonoids ranged from 41.21 to 146.06 mg of RU/g of extract which might play an important role in their stress adaptation. Halophytes like, *Atriplex tatarica*, *Camphorosma annua*, *Atriplex littoralis* are known to contain high amount of flavonoids [88]. Our results on higher flavone accumulation in *Sesuvium portulacastrum* under increasing salt stress indicate their possible antioxidant defense role as a secondary ROS-scavenging system as suggested by Fini et al. [89]. These studies suggested a possible role of flavonoids as antioxidants under stress condition in halophytes. However, this requires further studies to link specific flavonoid compounds in halophytes to their adaptation to salinity stress.

Lipids constitute the major components of biological membranes interfacing between the cell and the physical environment. Salinity disrupts membrane integrity and increases lipid peroxidation. We observed higher MDA content and lipid peroxidation in roots and leaves under salt stress. One of the compounds we isolated and characterized is a glycolipid (1-O-linolenyl-2-O-(palmitoyl)-3-O-galactopyranosyl glycerol) which showed a 1.2 fold higher accumulation under low salt treatment compared to HS and control. In LS, we observed higher MDA content which could possibly be linked to the higher accumulation of glycolipids in order to reduce membrane damage. In case of high salt stress, this increase may not be of much significance as membrane damage is irreversible. It is also indicative that diverse defensive mechanisms may be operative for the plant’s responses to salinity [90]. Salt tolerance in plants is strongly linked with their membrane lipid composition and especially with their galactolipid content, which is positively related to salt tolerance [27, 91]. Increased levels of galactolipids, particularly MGDG, contribute to the maintenance of organization and function of photosynthetic membranes, especially under stress conditions. Increased galactolipid content in leaves has been reported to be beneficial for maintaining chloroplast structure and function leading to enhanced salt tolerance [92, 93]. A distinct enhancement in 16:0/18:3 and 18:3/18:3 SQDG content was also noted in halophytes in order to in stabilize ATPase complexes and PSII activity under salt stress [31]. Our results on increased MGDG accumulation and increasing unsaturated fatty acids in *Sesuvium* plants therefore suggest that these metabolites could play a role in the adaptation of *Sesuvium* under salt stress.

## 5. Conclusions

Our results suggest that in the halophyte, *Sesuvium portulacastrum*, roots and leaves exhibit differential but coordinated responses to salt stress associated with the induction of high osmolytes, antioxidant enzymes and antioxidants like flavonoids and glycolipids to minimize the salt induced damage. Differential accumulation of potassium and sodium ions, and induction of temporal, transcriptional upregulation of *SOS1* and *NHX1* genes under high salinity in roots and leaves may be involved in controlling Na^+^ levels in *Sesuvium portulacastrum*. These findings help our understanding of the well-coordinated, salt adaptive mechanisms in halophytes that may be useful in improving crop plants.

## Supporting information

S1 Table Primers used for quantitative real-time PCR. (DOC)

S1 Info Details of the instrumentation used and operating conditions. (DOC)
