# Supplementary material for: Temporal and spatial changes in ion homeostasis, antioxidant defense and accumulation of flavonoids and glycolipid in a halophyte Sesuvium portulacastrum (L.) L
Source: PLoS One. 2018 Apr 11;13(4):e0193394. doi: 10.1371/journal.pone.0193394 (PMC5894978; doi:10.1371/journal.pone.0193394)
Supplement: S1 Table — (DOC) [file pone.0193394.s001.doc]

**Supplementary Table 1:** Primers used for quantitative real-time PCR.

| **Target gene** | **Primer name** | **Sequence 5’-3’)** | **Amplicon (bp)** |
| --- | --- | --- | --- |
| *NHX 3* | NHX3-F | CCGGAGTTGTGATTCTGCTG | 181 |
|  | NHX3-R | AATGTACCCAGGGCTCCAAA |
| *V type proton ATPase* | vATPase-F | TGGAGATCGCTTGACCACTT | 179 |
|  | vATPase-R | CAGAGTCGCCTTCTAACCGA |
| *SOS 1* | SOS1-F | TGTCGCTTTGCTCATCATCG | 220 |
|  | SOS1-R | TGGACCGGCAAGTATAAGCA |
| *Tub* | Tub-F | CCACAATTTCCTTTGCAACAGT | 250 |
|  | Tub-R | ATAGGTGTAGCCCATGATGCTT |
