# Supplementary material for: Temporal and spatial changes in ion homeostasis, antioxidant defense and accumulation of flavonoids and glycolipid in a halophyte Sesuvium portulacastrum (L.) L
Source: PLoS One. 2018 Apr 11;13(4):e0193394. doi: 10.1371/journal.pone.0193394 (PMC5894978; doi:10.1371/journal.pone.0193394)
Supplement: S1 Info — (DOC) [file pone.0193394.s002.doc]

**Details of the instrumentation used and operating conditions**

Absorption maxima of individual compound were obtained by using UV-Vis spectrophotometer (UV-1700, Shimadzu, Japan). The samples were scanned from 200 to 600 nm wavelength and methanol was used as blank.

Fourier Transform Infrared Spectroscopy (FT-IR) spectra was obtained by placing sample on ZnSe 45◦ Attenuated Total Reflectance (ATR) crystal which is equipped with deuterated l-alanine doped triglycene sulphate (DLaTGS) detector with KBr beam splitter. Spectra was collected in the range of wave number 4000-650 cm^−1^ with resolution of 4 cm^−1^ and 40 scans were accumulated for each sample using Jasco spectra manager version 2 software. Sample analysis was performed in triplicates and the mean was used for further analysis. The background scans were obtained from blank surface of crystal before each sample so as to avoid contaminating peaks. After each sample, the surface of crystal was cleaned with distilled water and lint free tissue.

The electron spray ionization mass spectrometer (ESI MS) analysis was performed using micrOTOF-Q-II quadrupole-time-of-flight mass spectrometer (Bruker Daltonics, Bremen, Germany) having ESI source (Jaison et al., 2012). The stable isotopes were simulated by using Compass Isotope Pattern software (Bruker Daltonics). A neMESYS automatic syringe pump (Cetoni Korbussen, Germany) was used for sample introduction in to ESI source with a flow rate of 4 µl^-1^ min. Compass Data Analysis software in MS and MS/MS modes was used for data collection and, positive ion mode was used for ESI MS analyses with 3500 V plate potential and 4500 V capillary voltage. High purity Argon was served as collision gas for MS/MS and nitrogen as both sheath gas and auxiliary gas. MS conditions are- Capillary temperature 180^o^C, Flow rate of sheath gas- 45 arbitrary units (a.u.) and flow rate of auxiliary gas was 4 (a.u.).

Nuclear Magnetic Resonance (NMR) spectra were recorded using Brüker Biospin, Switzerland, Avance AV 800 spectrometer, at the National Facility for High Field NMR, TIFR, Mumbai, India as per RoyChoudhury et al. (2016) and Kim et al. (2010). About 16K data points and total echo time of 38 ms were taken from Carr–Purcell–Meiboom–Gill (CPMG) spin-echo spectra for each sample. Total 16384 data points (TD) were used for collection of 256 transients for each sample with 14000 Hz spectral width which resulted into an acquisition time of 0.58 s. The phase and base line correction of spectra was done offline using MestReNova version 7.1.0 (Mestrelab Research, Santiago de Compostela, Spain) software.
